# Biochar Enhances Nutrient Uptake, Yield, and *NHX* Gene Expression in Chinese Cabbage Under Salinity Stress

**DOI:** 10.3390/plants14172743

**Published:** 2025-09-02

**Authors:** Periyasamy Rathinapriya, Theivanayagam Maharajan, Tae-Jun Lim, Byeongeun Kang, Seung Tak Jeong

**Affiliations:** 1 Horticultural and Herbal Crop Environment Division, National Institute of Horticultural and Herbal Science, Rural Development Administration, Wanju-gun 55365, Republic of Korea or rathina.priya25@gmail.com (P.R.); taejun06@korea.kr (T.-J.L.); lilacast97@korea.kr (B.K.); 2 Division of Plant Molecular Biology and Biotechnology, Department of Biosciences, Rajagiri College of Social Sciences, Cochin 683104, India; maharajan@rajagiri.edu

**Keywords:** Biochar, Chinese cabbage, gene expression, NHX transporter, salinity stress, soil nutrient

## Abstract

Salinity is a major limiting factor for all food crops, mainly Chinese cabbage. This study aimed to investigate the effects of biochar (BC) on physiological, biochemical, and molecular responses of Chinese cabbage grown under salinity stress in an open field. We supplied three concentrations of BC (5, 10, and 15 t/ha) to the 200 mM NaCl salinity-stress-induced field, which enhanced physical and chemical properties of the soil. Under salinity stress, BC increased photosynthetic pigments and reduced proline and H_2_O_2_ contents. Notably, 5 t/ha BC boosted plant growth, biomass, and yield by >40% and inhibited ROS accumulation under salinity stress. BC also promoted the concentrations of various key micronutrients, particularly Fe and Zn, in Chinese cabbage under salinity stress, which may contribute to improving the nutrient content. BC under salinity stress significantly induced the expression of *NHX* family genes (*BoNHX1* and *BoNHX2*). Among these, the *BoNHX1* gene was found to be highly expressed in shoot and root tissues of Chinese cabbage grown under salinity stress with BC. Identification of this key candidate gene will lay the groundwork for further functional characterization studies to elucidate its role under salinity stress with BC. This study comprehensively analyzes the physiological, biochemical, and molecular impacts of BC application in Chinese cabbage under salinity stress. This study found that the application of 5 t/ha significantly improved various physiological and biochemical traits of Chinese cabbage under salinity stress compared to the other treatments. The outcome of this study provides novel insights into the bioprotective role of BC, offering a valuable foundation of organic supplements for farmers while also highlighting potential research directions for enhancing crop resilience and productivity in economically important crops.

## 1. Introduction

Chinese cabbage (*Brassica pekinensis* L.) is a nutritionally dense vegetable that plays a crucial role in human health and dietary needs worldwide. It is rich in essential nutrients and minerals, including vitamin A (223 µg/100 g), vitamin C (45 mg/100 g), vitamin K (45.5 µg/100 g), folate (66 µg/100 g), Ca (105 mg/100 g), Fe (0.8 mg/100 g), Mg (19 mg/100 g), P (37 mg/100 g), K (252 mg/100 g), and ß-carotene (2681 µg/100 g), which helps in maintaining daily nutritional requirements for humans [1]. These nutrients in Chinese cabbage contribute to various health benefits like promoting bone density, blood clotting, curing constipation, dental strength, and improving vision; helping in regulating fluid balance; preventing obesity; supporting the cardiovascular system; enhancing immune function; and providing antioxidant defense due to its high nutritional profile [2,3]. Furthermore, its consumption is essential for DNA synthesis and repair during pregnancy. Chinese cabbage is extensively cultivated in Europe, the Mediterranean, and East Asia, especially in Korea, China, and Japan, due to its significant health benefits [4]. It is a key ingredient in kimchi, an essential and staple side dish at most meals in South Korea [5]. Due to their very short growing season and moderate cold tolerance abilities, fresh cabbage can be produced all year round in the open field [6]. However, for many years, various biotic (bacterial and fungal diseases) and abiotic stresses (especially drought, salinity, heat, and heavy metal toxicity) have controlled the cultivation and yield of Chinese cabbage [7].

Significant research has concentrated on enhancing the Chinese cabbage cultivars’ resistance to biotic stressors [8]. However, only a few researchers work on improving the growth and yield of Chinese cabbage against abiotic stresses (particularly salinity stress). Of the various abiotic stresses, it is highly sensitive to salinity stress, which leads to a reduction in germination rate, stunted growth, decreased yield, and severely diminished nutritional quality [9]. Therefore, farmers utilized inorganic fertilizers to enhance the nutritional content of the soil, promoting the growth of Chinese cabbage on salinity-stressed agricultural land [10]. Sometimes farmers use excessive amounts of fertilizers in the soil without adequate knowledge, which pollutes the ecosystem (especially soil) and reduces Chinese cabbage growth due to nutrient toxicity/imbalance [11,12]. Therefore, adopting eco-friendly management techniques is crucial to enhance Chinese cabbage growth and preserve the environment. Hence, developing salinity stress-tolerant Chinese cabbage cultivars could greatly improve their productivity in saline soils and support sustainable agriculture.

Soil salinity is a widespread agronomic issue that affects over 1000 million hectares of land universally, accounting for about 20% of the world’s irrigated farmlands, leading to significant declines in sustainable crop production [13,14,15]. Salinity stress diminishes crop yield by 5–50%, primarily due to ionic toxicity, nutrient imbalances, and osmotic stress [16]. The excessive accumulation of Na^+^ in the soil interferes with plant water uptake, disrupts nutrient balance, degrades soil quality, and severely impairs the growth, yield, and nutritional quality of crops [13]. In addition, excessive Na^+^ competes with essential nutrients like potassium, leading to nutrient deficiencies and physiological disorders in plants [17]. Mitigating soil salinity stress is urgently needed to enhance soil quality and ensure sustainable agricultural productivity and food security. To cope with these adverse effects, various strategies have been employed to improve crop performance under salinity stress. Among them, BC application has emerged as a promising eco-friendly approach to ameliorating soil salinity without affecting any soil properties.

BC, a pyrolyzed carbonaceous material, has been shown to enhance soil structure, pH, OM, EC, and water retention, as well as reduce the mobility of toxic ions such as Na^+^ [18,19,20]. Its high surface area and porosity enhance nutrient retention and reduce leaching, particularly in sandy or degraded soils [21,22]. BC improves cation exchange capacity by replacing harmful Na^+^ with beneficial K^+^ and Mg^2+^, thereby restoring nutrient balance and lowering the Na^+^/K^+^ ratio [23,24,25]. Additionally, BC improves the availability and retention of nutrients in soil by raising the cation exchange capacity and carbon stability, especially in soils with low OM [26]. It also improves plant growth, leaf function, and yield by making nutrients more available and encouraging beneficial microbial activities, which makes the soil healthier [27,28]. Along with that, it lowers the Na^+^/K^+^ ratio, keeps LWC high, and boosts plant shoot and root growth in tomato (*Solanum lycopersicum*), potato (*Solanum tuberosum*), and soybean (*Glycine max*) plants that are stressed by salinity [29,30,31]. Similarly, BC increases plant stomatal conductance, photosynthesis, and transpiration rate; regulates osmotic potential; and reduces proline content, superoxide dismutase activity, abscisic acid, and soluble sugar in various food crops such as sorghum (*Sorghum bicolor*) [32], mung bean (*Vigna radiata*) [33], maize (*Zea mays*) [34], brinjal (*Solanum melongena*) [35], cabbage (*Brassica oleracea*) [36], and wheat (*Triticum aestivum*) [37,38]. Additionally, it improves the soil’s quality and helps plants deal with salt stress by lowering their uptake of Na^+^ and increasing their ability to handle stress [39]. However, many horticultural and economically important plants, including Chinese cabbage, have not undergone extensively studies of the role of BC in physiological, biochemical, and molecular responses under salinity stress. All of this, when analyzed in detail in a single plant under salinity stress, will help researchers learn about the BC mechanism.

Plants alleviate salinity stress through various salt stress-responsive and tolerant genes that regulate ion homeostasis, osmotic balance, and stress response pathways [40]. Several salinity stress-responsive genes have been identified and characterized in various plants [41]. For example, salt overly sensitive (*SOS*), high-affinity potassium transporters (*HKTs*), Na^+^/H^+^ antiporters (*NHX*), and antioxidant defense genes play key roles in enhancing salinity tolerance [42]. *SOS* genes (*SOS1*, *SOS2*, *SOS3*) regulate ion homeostasis under salt stress [43], while *HKT* family members maintain Na^+^ and K^+^ balance. *AtHKT1;1* and *AtHKT1;5* in Arabidopsis reduce Na^+^ toxicity by excluding Na^+^ from shoots [44]. Antioxidant genes such as ascorbate peroxidase (*APX*), superoxide dismutase, catalase, and peroxidase, along with transcription factors like *WRKY*, *DREB*, *NAC*, and *ERF*, help regulate ROS and improve salt stress resilience [45,46]. The activities of all the transporters and transcription factors mentioned above counteract Na^+^ accumulation and eliminate its detrimental effect in the cytosol. Compared to the other genes, *NHX* family genes help keep the balance of ions inside cells by placing extra Na^+^ into vacuoles [47]. This prevents the excess Na^+^ from harming plant cells. Similarly, *NHX* family genes play an important role in conferring salinity tolerance by ensuring that cytoplasmic Na^+^ concentrations remain low, which is critical for maintaining normal cellular functions [48].

NHX proteins belong to the cation/proton antiporter 1 superfamily, and most *NHX* family members have around 10–12 transmembrane domains [49]. They are primarily located in plasma membranes, vacuoles, and endosomes [48]. The *NHX* gene was first identified in the root tip of barley in 1976 [50]. Since then, more than seven *NHX* members have been identified in plants. For example, a total of 8 *NHX* genes (*AtNHHX1* to *AtNHX8*) in Arabidopsis, 30 in wheat (*TaNHX1* to *TaNHX*30) [51], 9 in soybean (*GmNHX1* to *GmNHX9*) [52], 7 in tomato (*SlNHX1* to *SlNHX7*) [53], and 5 in sugar beet (*BvNHX1* to *BvNHX5*) [54] have been identified from their respective plant genomes, and their expression patterns in various tissues under salinity stress. Apart from these plants, around five to eight *NHX* family genes have been identified in several cereals, vegetables, and fruits.

It is noteworthy that *NHX* family genes have not yet been identified for Chinese cabbage. The completely annotated genome sequences for cabbage were published in 2014 [55], but no one has yet begun to identify the *NHX* family genes for this plant. In this study, we evaluated the physiological and biochemical responses of Chinese cabbage to BC application under salinity stress in an open field. We also emphasize the importance of optimizing BC concentration, as it plays a critical role in improving plant growth, nutrient availability, and stress tolerance. Then, we aimed to investigate the expression profile of *NHX* family genes in different tissues of Chinese cabbage grown under salinity stress with a supply of BC. This research not only advances our understanding of the molecular mechanisms underlying salinity stress in Chinese cabbage but also offers practical solutions to improve its cultivation in saline-prone areas, thereby supporting global efforts toward sustainable agricultural practices and food security.

## 2. Results

### 2.1. Effects of BC on Soil Parameters Under Salinity Stress

**Figure 1:**
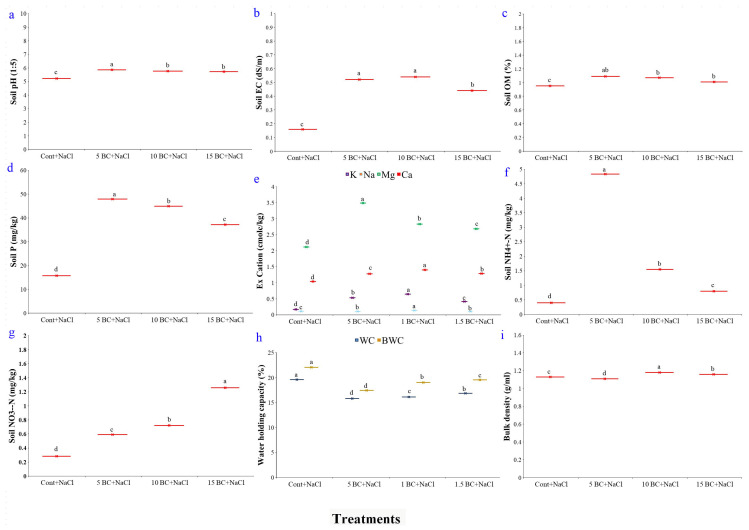
Effects of BC on the physical and chemical properties of soil collected from salinity-induced field. All the physical and chemical properties of soil were collected from BC-treated field. The graphs of pH, EC, OM, P_2_O_5_, exchangeable cation, NH^4+^N, NO^3−^N, WHC, and BD were designated as (**a**–**i**), respectively. The values are the mean ± standard deviation (SD) of independent triplicates (*n* = 12). All values obtained from each trait were significantly different responses under various levels of BC and control (*p* < 0.05) based on a Tukey’s post-hoc test.

The physical and chemical properties of soil were assessed under salinity stress with and without BC supplementation (Figure 1a–i). The results indicated that BC application significantly improved soil structure and nutrient availability compared to the control treatments. Additionally, the presence of BC helped mitigate the adverse effects of salinity stress, enhancing overall soil health and resilience. Results indicated no significant differences in soil properties between the three BC treatments. However, significant variations were observed for both physical and chemical properties of soil with and without BC amendments. With 5 t/ha of BC during salinity stress, the soil pH rose to about 5.8, while the control had a pH of 5.2, showing a small increase in alkalinity (Figure 1a). Notably, all BC treatments increased soil EC compared to the control, with 5 t/ha reaching 0.52 dS/m and 15 t/ha at 0.44 dS/m (Figure 1b). In contrast, the control had the lowest soil EC (0.16 dS/m). The OM content decreased marginally to 1.09% (5 t/ha), 1.07% (10 t/ha), and 1.01% (15 t/ha) with increasing BC application, whereas available P increased dramatically, particularly at 5 t/ha (47.94 mg/kg) and 10 t/ha (44.89 mg/kg) compared to the control (15.71 mg/kg), indicating enhanced P availability in the soil (Figure 1b–d).

Figure 1e presents the concentrations of exchangeable cations, such as K, Na, Mg, and Ca. Soil amended with BC had the highest amount of K, measuring 0.65 cmolc/kg at 10 t/ha soil. However, as the BC levels increased, the extractable K dropped to 0.42 cmolc/kg at 15 t/ha, while the control soil showed 0.17 cmolc/kg. The Na^+^ content gradually increased from 0.11 cmolc/kg in the control to 0.14 cmolc/kg at 10 t/ha, showing slight variations across BC treatments. The result suggests a moderate accumulation effect, likely due to BC’s cation exchange capacity. Mg levels showed an initial increase with BC application (up to 3.49 cmolc/kg at 5 t/ha) but decreased at higher application rates. Similarly, the Ca content of the soil increased at 10 t/ha (1.40 cmolc/kg), whereas a reduction was noticed at 15 t/ha and in the control soil. These results clearly demonstrated that moderate BC levels retain nutrients, modifying the soil’s cation exchange capacity, which is highly beneficial for plant growth. The soil with the highest levels of NH_4_^+^-N (4.83 mg/kg) and NO_3_^−^-N (0.59 mg/kg) was 5 t/ha (Figure 1f,g). Higher BC rates (1.0 and 15 t/ha) slightly increased N, especially NO_3_^−^-N. This suggests that N is more available and NO_3_^−^-N becomes more important at higher BC concentrations. We measured WC, BWC, and BD in field soil to evaluate the effect of BC on water-holding capacity. Control soil exhibited higher WC (19.62%) and BWC (22.03%) than BC treatments. BC treatments at 5 t/ha reduced the WC by 15.84% and BWC by 17.45% compared to the control. In contrast, higher levels of BC (1.0 and 15 t/ha) slightly raised WC to 16.15% and 16.87% and BWC to 19.06% and 19.57%, respectively (Figure 1h). BD showed minimal changes, with the 5 t/ha treatment showing 1.11 g/mL (1.77% less than the control), while 1.0 and 15 t/ha increased BD to 1.18 g/mL and 1.16 g/mL, respectively (Figure 1i). These results suggest that BC, particularly at lower rates, reduces soil water retention without notably altering BD, potentially improving water infiltration and soil aeration.

### 2.2. Effects of BC on Biochemical Traits of Chinese Cabbage Under Salinity Stress

**Figure 2:**
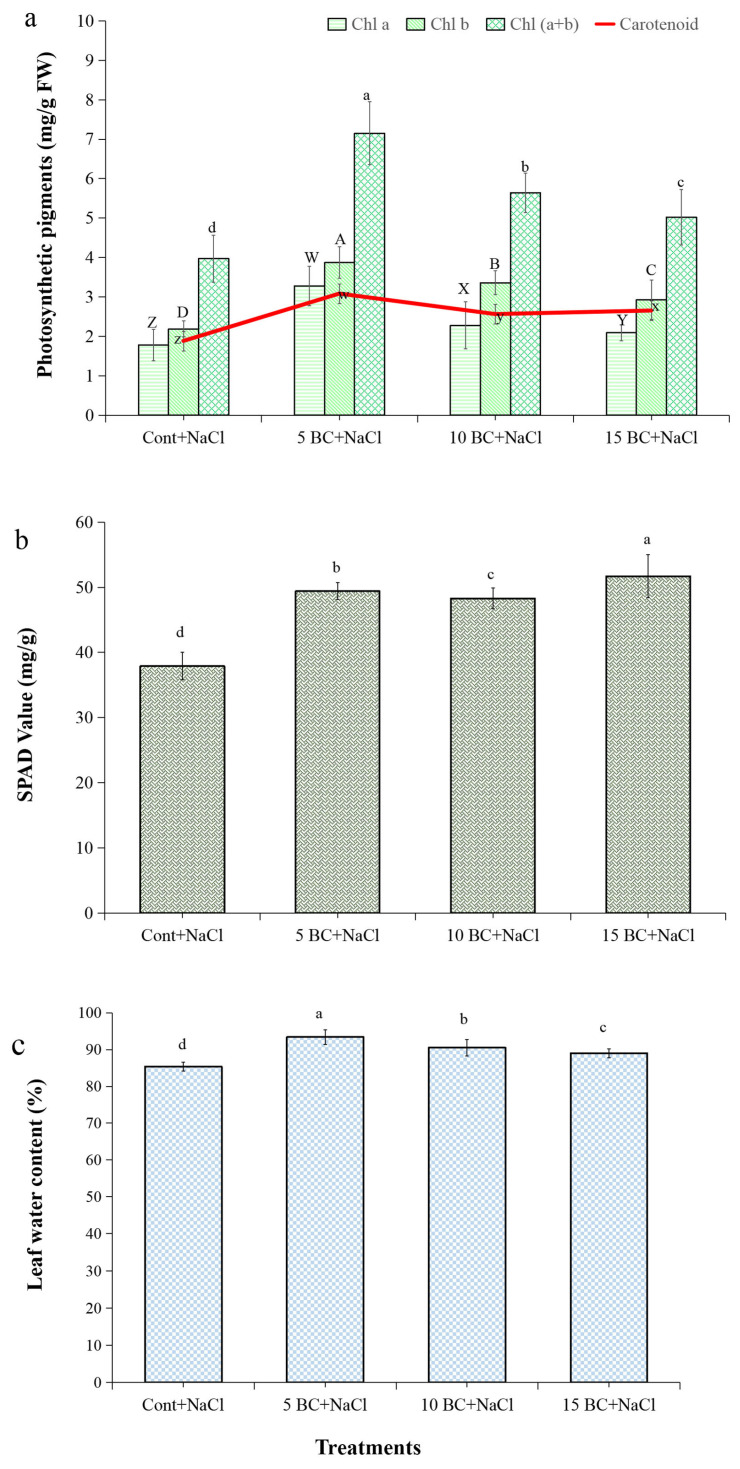
Effect of salinity stress and BC on photosynthetic pigments and leaf water content in Chinese cabbage. Chlorophyll a, b, and a + b; carotenoid; SPAD; and leaf water contents were analyzed in the 90-day-old leaves of Chinese cabbage grown under both control and salinity stress with three different concentrations of BC. The values are the mean ± standard deviation (SD) of independent triplicates (*n* = 12). All values obtained from each trait were significantly different responses under various levels of BC and control (*p* < 0.05) based on a Tukey’s post-hoc test. (**a**–**c**) denoted photosynthetic pigments, SPAD values, and leaf water contents, respectively. Statistically significant analyses were performed separately for each trait between the control and BC treatments.

Under salinity stress, all BC-grown plants showed improvements in both chlorophyll and carotenoid values (Figure 2a). In the 5 t/ha BC treatment, chlorophyll a was 84.3%, chlorophyll b was 76.7%, total chlorophyll a + b was 50.4%, SPAD was 30.3%, and carotenoids were 63.8%, all of which were higher than in the control group. The SPAD value in control plants was 37.9 mg/g, and in the 15 t/ha BC treatment, it was 51.7 mg/g (Figure 2b). This result indicates that BC increased SPAD by 26.6% compared to the control.

These findings demonstrate BC’s effectiveness in improving chlorophyll content and SPAD values, particularly under salinity stress. Adding BC to the soil at a concentration of 5 t/ha might have lessened the negative effects of salt on the amounts of chlorophyll and carotenoids in Chinese cabbage. The analysis of LWC in Chinese cabbage under salinity stress is crucial for understanding plant resilience, growth efficiency, and yield optimization. The data demonstrated that BC treatments positively influenced LWC under salinity stress, as shown in Figure 2c. The 5 t/ha BC treatment showed the highest LWC at 93.4%, followed by 90.5% at 10 t/ha and 89.0% at 15 t/ha, representing an 11.1% increment compared to the control.

**Figure 3:**
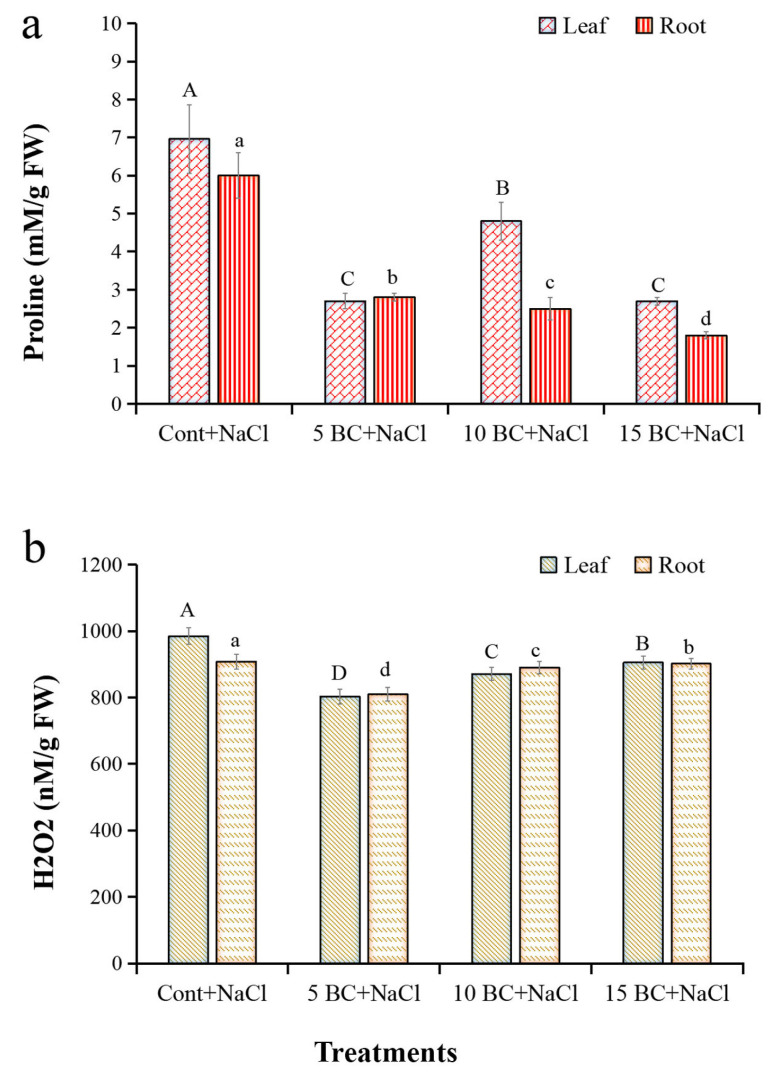
Effect of BC on proline and H_2_O_2_ analysis of Chinese cabbage grown under salinity stress. Proline and H_2_O_2_ contents were analyzed in 90-day-old leaves of Chinese cabbage grown under both control and salinity stress with three different concentrations of BC. All values obtained from each trait were significantly different responses under various levels of BC and control (*p* < 0.05) based on a Tukey’s post-hoc test. (**a**,**b**) denoted proline and H_2_O_2_ contents, respectively. Statistically significant analyses were performed separately for each trait between the control and BC treatments.

The result suggests that plants grown without BC exhibited the least LWC, but 5 t/ha BC significantly enriched the water retention in Chinese cabbage under salinity stress. The increase of proline and H_2_O_2_ in plants is generally considered the oxidative burst that activates the plant’s antioxidant defense system in response to salinity stress. The proline and H_2_O_2_ generation exhibited significant differences between the BC-treated and the control plants. The 5 t/ha BC treatment significantly affected the proline (leaf 2.7 mM/g FW, root 2.8 mM/g FW) and H_2_O_2_ (leaf 802.5 nM/g FW, root 809.6 nM/g FW) contents compared to the control (Figure 3a,b).

**Figure 4:**
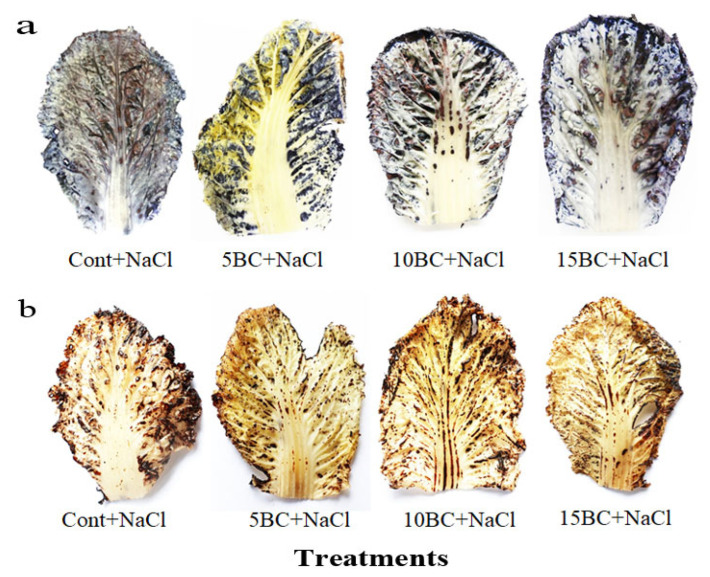
Histochemical detection of in situ analysis of superoxide anion (O_2_^−^) using NBT and H_2_O_2_ using DAB staining in salinity induced Chinese cabbage grown under BC. (**a**) NBT detection of O^2−^; (**b**) DAB staining of H_2_O_2_ in salinity induced in 90-day-old leaves of Chinese cabbage grown under control and three different concentrations of BC. Here, control, 5 BC+NaCl, 10 BC+NaCl, and 15 BC+NaCl (Left to right).

The 15 t/ha BC-treated Chinese cabbage showed a reduced concentration of proline in its leaves (2.7 mM/g FW) and roots (1.8 mM/g FW). Similarly, in 10 t/ha BC, leaves and roots significantly decreased H_2_O_2_ generation by 0.8- and 0.9-fold less than those seen in the control plants under salinity, respectively. Using DAB and NBT staining, we looked at how applying BC changed the ROS removal process in Chinese cabbage that was under salt stress. Control plants exhibited intense staining, indicating elevated ROS levels compared to those treated with BC (Figure 4). Plants treated with BC at concentrations of 5, 10, and 15 t/ha showed a marked reduction in staining intensity. This reduction in the accumulation of O_2_¯ in NBT (Figure 4a) and H_2_O_2_ in DAB (Figure 4b) was most pronounced with the 5 t/ha BC treatment. These results show that applying BC effectively reduces oxidative stress by changing the homeostasis of ROS. This implies that it could play a crucial role in aiding Chinese cabbage’s recovery from the harm resulting from salty soil.

### 2.3. Effects of BC on Chinese Cabbage Phenotypic Traits

**Figure 5:**
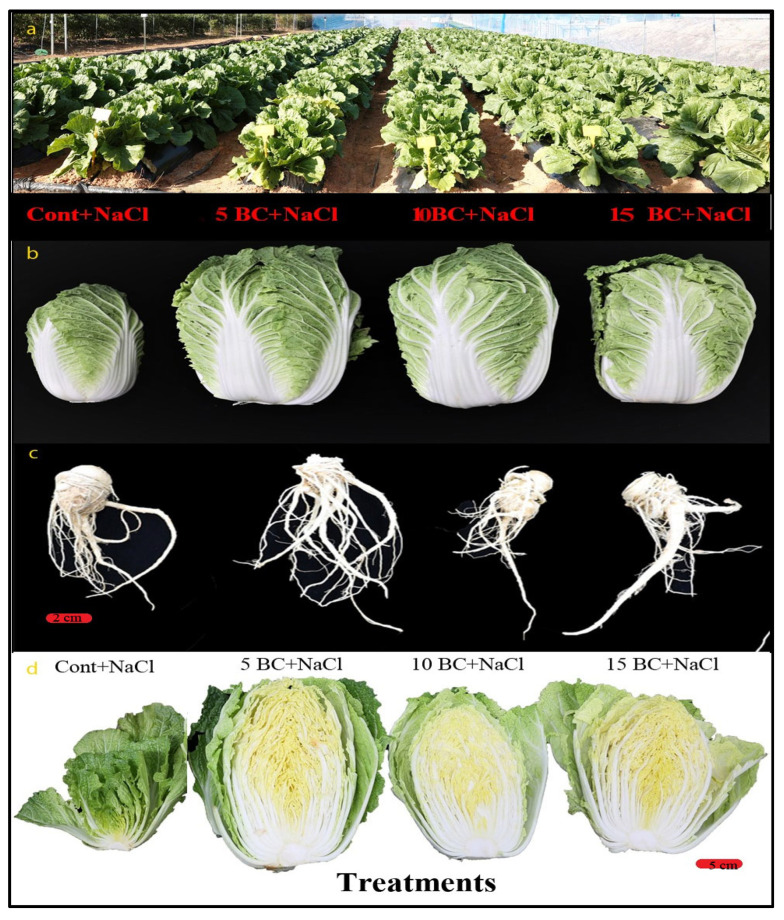
Effect of BC application on 90-day-old Chinese cabbage growth under salinity stress. (**a**) Open field view showing the growth performance of Chinese cabbage in treatments with control (Cont+NaCl), 5 BC+NaCl, 10 BC+NaCl, and 15 BC+NaCl (left to right). (**b**) Representative Chinese cabbage heads harvested from each treatment groups. (**c**) Root systems of Chinese cabbage under different treatments (*bar* = 2.0 cm). (**d**) Cross-sectional views of Chinese cabbage heads illustrating internal quality grown under control and with three difference concentrations of BC (*bar* = 5.0 cm).

The role of BC in enhancing salinity stress tolerance was evaluated by measuring the growth parameters of 90-day-old Chinese cabbage plants grown under three different BC concentrations and control upon salinity stress (Figure 5). The 5 t/ha treatment produced the longest LL (49.9 cm) and RL (25.6 cm). The control plants had significantly lower LL (28.9 cm) and RL (18.6 cm), indicating a substantial increase in plant growth with BC applications. Under control, the LL and RL decreased by 42% and 27.3%, respectively, compared to 5 t/ha BC. LL values for the 10 t/ha and 15 t/ha treatments were 41.1 cm and 37.3 cm, and RL 21.1 cm and 20.9 cm, respectively. Compared to these two BC treatments, control plants reduced their LL and RL by >10%.

**Figure 6:**
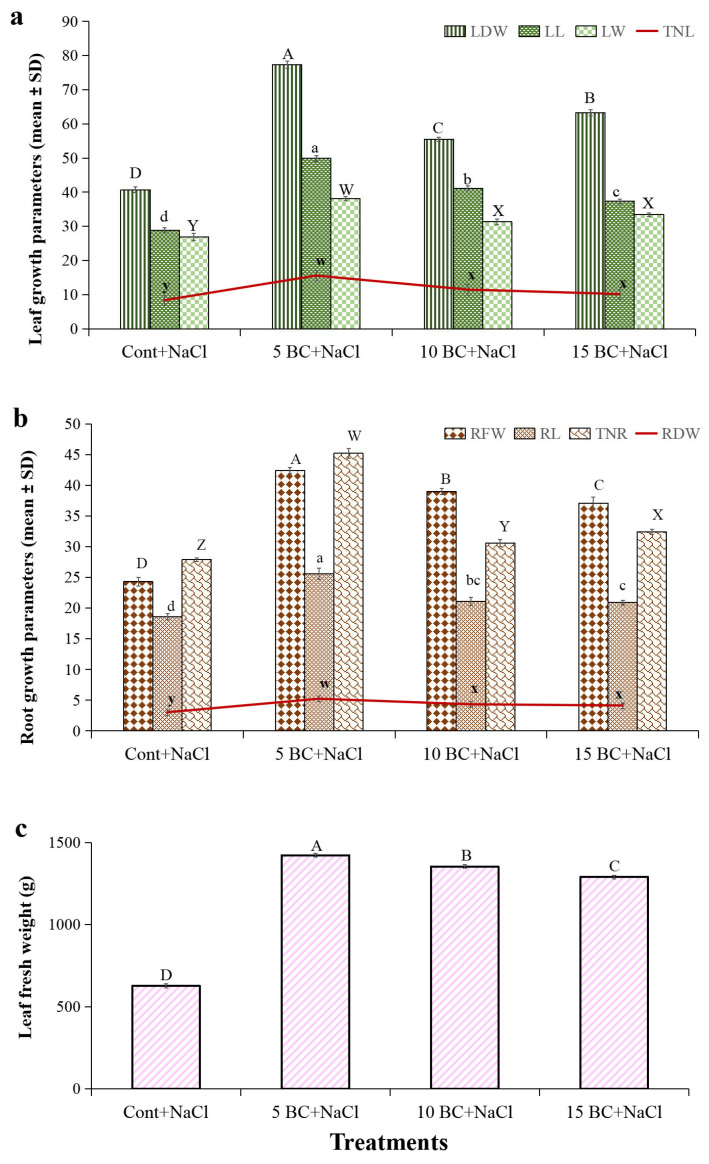
Effect of BC treatment on the growth parameters of 90-day-old Chinese cabbage plants exposed to salinity stress. (**a**) Leaf growth parameters. (**b**) Root growth parameters. (**c**) Leaf fresh weights were analyzed in 90-day-old Chinese cabbage grown under both control and salinity stress with three different concentrations of BC. All values obtained from each trait were significantly different responses under various levels of BC and control (*p* < 0.05) based on a Tukey’s post-hoc test. Statistically significant analyses were performed separately for each trait between the control and BC treatments.

Chinese cabbage produced approximately 9 TNL and 28 TNR in the control, while the BC-treated plants produced over 11 TNL (Figure 6a). Similarly, >15 TNL and >45 TNR were observed in 5 t/ha BC, and >11 TNL and >30 TNR were observed in 10 t/ha BC, respectively. The LW followed a similar pattern, with the control plants exhibiting an LW of 26.9 cm. In contrast, the 5 t/ha BC treatment significantly increased the LW to 38.1 cm. LW increased by 29.3% at 5 t/ha BC compared to the control. The 10 t/ha BC treatment showed an LW of 31.3 cm, while the 15 t/ha treatment resulted in an LW of 33.4 cm (Figure 6a). The 5 t/ha BC treatment recorded the highest LFW of 142.1 g, followed by 10 t/ha (1353.4 g) and 15 t/ha (1288.8 g). The RFW and LDW also increased significantly with 5 t/ha BC. Control plants exhibited lower LFW (626.9 g), RFW (24.3 g), and LDW (40.7 g) compared to the BC-supplemented plants. The higher concentration of BC (15 t/ha) resulted in a decreased LFW of 1288.8 g, an RFW of 37.1 g, and an RDW of 4.1 g (Figure 6b,c). These findings demonstrate that BC, particularly at the 5 t/ha rate, significantly boosts the biomass and growth recovery of Chinese cabbage plants under high salinity stress. This enhances the plants’ ability to withstand the negative impacts of salinity stress.

### 2.4. Influence of BC upon Salinity Stress in Yield Parameters of Chinese Cabbage

**Figure 7:**
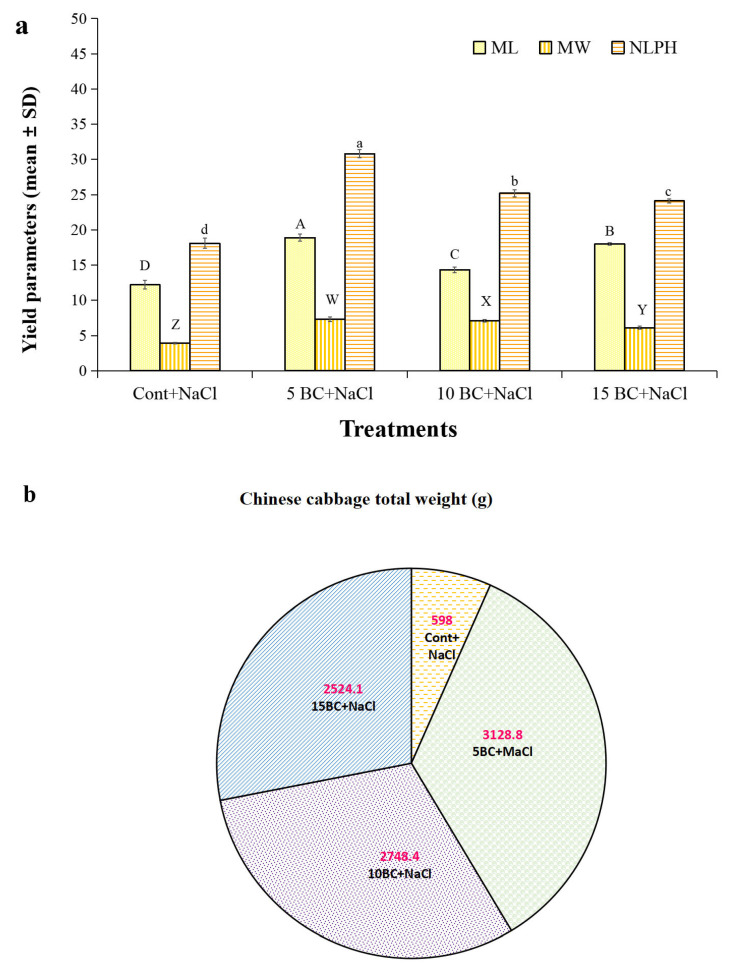
Effect of BC treatment on the yield parameters of 90-day-old Chinese cabbage plants exposed to salinity stress. (**a**) Yield parameters. (**b**) Total weight were analyzed in 90-day-old Chinese cabbage grown under both control and salinity stress with three different concentrations of BC. The values are the mean ± standard deviation (SD) of independent triplicates (*n* = 12). All values obtained from each trait were significantly different responses under various levels of BC and control (*p* < 0.05) based on a Tukey’s post-hoc test. Statistically significant analyses were performed separately for each trait between the control and BC treatments.

Salinity stress significantly altered the ML, MW, NLPH, and TW of the Chinese cabbage head, as we observed. Compared to the BC-treated plants, the control reduced NLPH (18.1 cm), ML (12.2 cm), and MW (3.9 cm) (Figure 7a). In 5 t/ha BC, the highest ML (18.9 cm), MW (7.3 cm), and NLPH (30.8) were also at 1.0 and 15 t/ha BC, noted with increased ML, MW, and NLPH over the control. BC treatment (5 t/ha) significantly enhanced the NLPH, ML, and MW by 41.2%, 35.4%, and 46.5%, respectively, compared to the control. The total yield weight showed the greatest improvement with the BC application (Figure 7b). The control plants yielded a TW of 598.0 g, whereas the 5 t/ha BC treatment yielded the highest yield (3128.8 g; TW), indicating an 80.8% increase compared to the control. The 10 t/ha BC treatment increased TW by 78%, and the 15 t/ha BC treatment increased TW by 76% compared to the control (Figure 7b). It is important to note that 5 t/ha BC increased TW by 12.7% and 19% more than the 1.0 and 15 t/ha BC treatments, respectively. Despite these marginal reductions, both BC treatments resulted in significantly higher TW compared to the control. Higher concentrations of BC 1.0 and 15 t/ha in this study also improved yield under salinity stress; however, we noticed that the 5 t/ha treatment was the most efficient in enhancing Chinese cabbage yield. The control plants exhibited the lowest TW, emphasizing the importance of BC supplementation to significantly augment crop yield under salinity conditions.

### 2.5. Analysis of Essential Macro- and Micronutrient Contents of Chinese Cabbage Grown Under Salinity Stress with a Supply of BC

**Figure 8:**
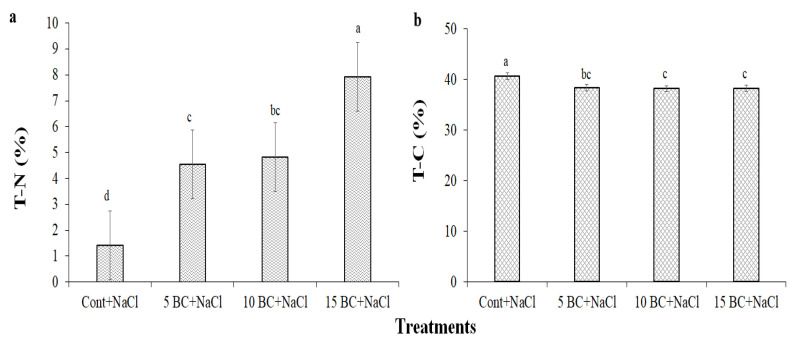
Effect of BC treatment on T-N and T-C contents of 90-day-old Chinese cabbage plants exposed to salinity stress. (**a**) T-N and (**b**) T-C were analyzed in 90-day-old Chinese cabbage grown under both control and three different concentrations of BC upon salinity stress. The values are the mean ± standard deviation (SD) of independent triplicates (*n* = 12). All values obtained from each trait were significantly different responses under various levels of BC and control (*p* < 0.05) based on a Tukey’s post-hoc test.

In this study, BC amendment under salinity significantly influenced the essential nutrient content of Chinese cabbage. Salinity stress drastically decreased the macro- and micronutrients in the leaves, but BC applications comparatively improved them. Salinity treatment significantly reduced the T-N and T-C content in the Chinese cabbage plants. The control plants exhibited the lowest T-N content (1.42%), while T-N levels increased at 5 t/ha and 10 t/ha BC upon salinity treatment. Interestingly, T-N content reached the highest level, 7.93%, at 15 t/ha BC compared to other BC treatments (Figure 8a). On the other hand, the T-C content showed a slight decrease from 40.6% at control to 38.3% at 5 t/ha and showed minimal variation at higher BC levels (Figure 8b).

**Figure 9:**
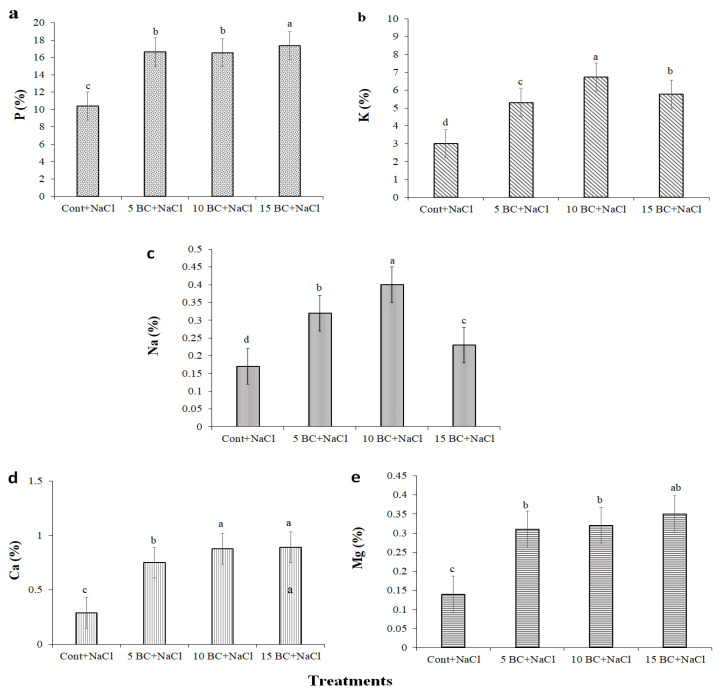
Effect of salinity stress on the macronutrient contents of Chinese cabbage grown under control and three difference concentrations of BC. The graphs of P, K, Na, Ca, and Mg were designated as (**a**–**e**), respectively. All values obtained from each trait were significantly different responses under various levels of BC and control (*p* < 0.05) based on a Tukey’s post-hoc test. Statistically significant analyses were performed separately for each trait between the control and BC treatments.

The BC amendment potentially augmented the macronutrient composition of Chinese cabbage, such as P, K, Ca, Mg, and Na, which enhanced plant resilience under saline conditions. P content increased sequentially in all BC treatments while noted as highest at 10 t/ha (17.34%) (Figure 9a). K content exhibited (5.30%) in 5 t/ha, (6.73%) in 10 t/ha, and at 15 t/ha (5.77%) (Figure 9b). Na levels increased by 5 t/ha (0.32%) and 10 t/ha (0.40%); however, they decreased to 0.23% at 15 t/ha (Figure 9c). BC amendment significantly enriched the plant Ca and Mg contents under salinity stress. In particular, plants grown in 15 t/ha BC exhibited a 0.89% increase in Ca and a 0.35% augmentation in Mg content compared to the control, respectively. The promoting effects of all three BC treatments on Ca and Mg absorption were significantly higher than the control (Figure 9d,e).

**Figure 10:**
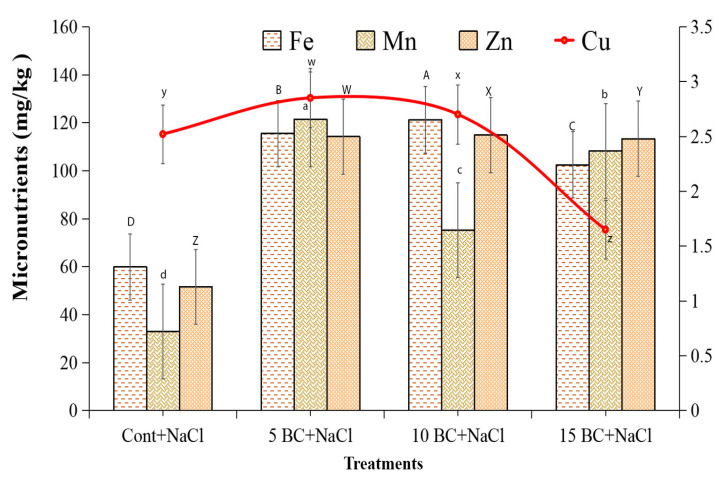
Effect of BC on the micronutrient contents of Chinese cabbage grown under control and three difference concentrations of BC upon salinity. The Fe, Mn Zn, and Cu were analyzed in 90-day-old Chinese cabbage grown under both control and three different concentrations of BC under salinity stress. All values obtained from each trait were significantly different responses under various levels of BC and control (*p* < 0.05) based on a Tukey’s post-hoc test. Statistically significant analyses were performed separately for each trait between the control and BC treatments.

BC treatments had a notable impact on micronutrient levels in Chinese cabbage under salinity stress. Specifically, BC treatments significantly enriched Fe and Zn levels. The 10 t/ha BC recorded the highest Fe level at 121.2 mg/kg, indicating a substantial increase of 59.8 mg/kg compared to the control. Fe content in leaves was almost 50.6% higher in 10 t/ha BC than in the control. Results showed that 5 t/ha BC and 15 t/ha BC increased Mn content (121.5 and 108.2 mg/kg, respectively) compared to the control (32.97 mg/kg) (Figure 10). In addition, Cu content exhibited minor variations, with a marginal rise at 5 t/ha BC and 10 t/ha BC. When plants were exposed to salinity stress, they took in 2.2 times more Zn than control plants did at all BC concentrations. They also took in a lot more Zn than Fe, Mn, and Cu micronutrients (Figure 10). The results showed that adding moderate amounts of BC to Chinese cabbage increased the availability of important micronutrients like Fe, Mn, and Zn when the plant was under salt stress. This procedure could have improved the plant’s health and growth.

### 2.6. Expression Pattern of NHX Family Genes in Leaf and Root Tissues of Chinese Cabbage Grown Under Salinity Stress with Different Combinations of BC

A total of two *BoNHX* family genes were used for this experiment because there are currently only two NHX family genes in the Brassica genome. The details of *BoNHX* genes, including genomic location, length of nucleotide and protein sequences, chromosome number, MW, pI, number of TMDs, and subcellular localizations, are listed in Supplementary Table S1.

**Figure 11:**
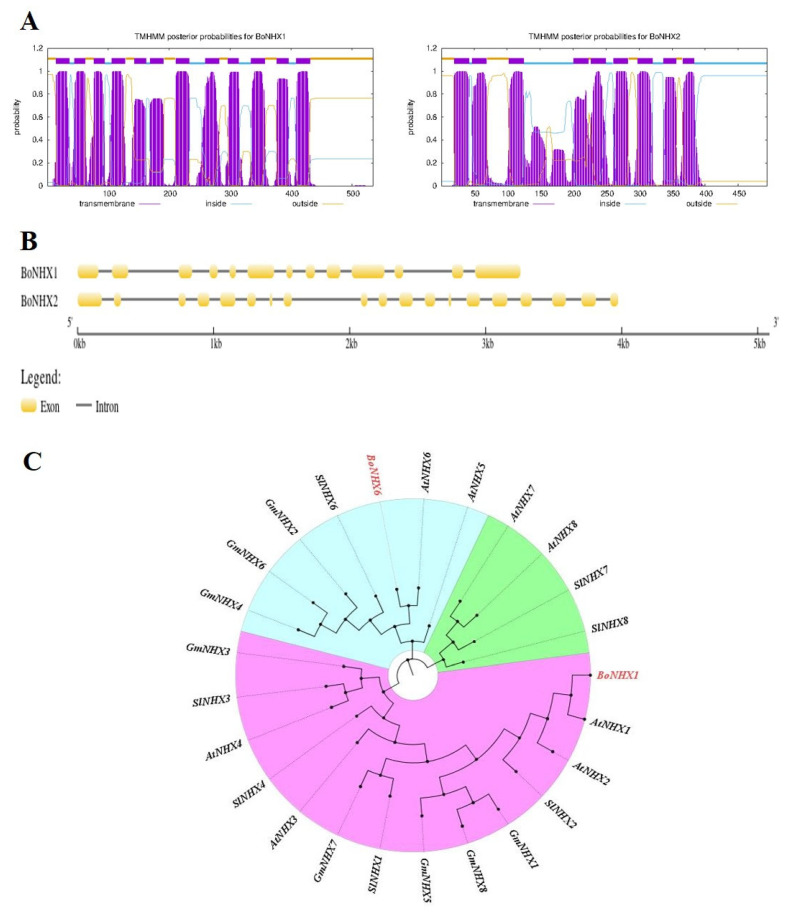
Insilco analysis of Chinese cabbage NHX genes/proteins. A phylogenetic tree of the Chinese cabbage family proteins was constructed with those of other *Poaceae* family members such as Arabidopsis, soybean, and tomato. (**A**) The tree was constructed using 25 potential NHX family transporter proteins from four plant species, including two Chinese Cabbage NHX family proteins, by MEGA version 6 software using the maximum likelihood method based on the Jones–Taylor–Thornton matrix-based model with 1000 bootstrap replicates. The phylogenetic tree was visualized and edited using the FigtreeV1.4.2 tool. The BoNHX family proteins of Chinese cabbage are indicated in red. (**B**) The number of TMDs was predicted for BoNHX family proteins of Chinese cabbage using the TMHMM server v.2.0, an online-enabled tool. (**C**) The full-length genomic sequences of *BoNHX* were used to determine the intron/exon distribution by gene structure display server program.

The nucleotide lengths of the two *BoNHX* genes ranged from 1608 (*BoNHX1*) to 1485 (*BoNHX2*). The length of BoNHX proteins ranged from 535 *(BoNHX1*) to 494 (*BoNHX2*). The predicted MW of BoNHX proteins ranged from 59.35 (BoNHX1) to 54.77 (*BoNHX2*) kDa, and the pI ranged between 7.62 (*BoNHX1*) and 6.11 (*BoNHX2*). All BoNHX proteins possess 9–11 TMDs and would be localized to the plasma membrane. The intron number in the *BoNHX* family genes varies greatly; *BoNHX1* has 12 introns, and *BoNHX2* has 18 introns (Figure 11A,B). To investigate the evolutionary relationships among the identified BoNHX proteins, we constructed a phylogenetic tree using a total of 25 protein sequences, including two BoNHX proteins (Figure 11C).

**Figure 12:**
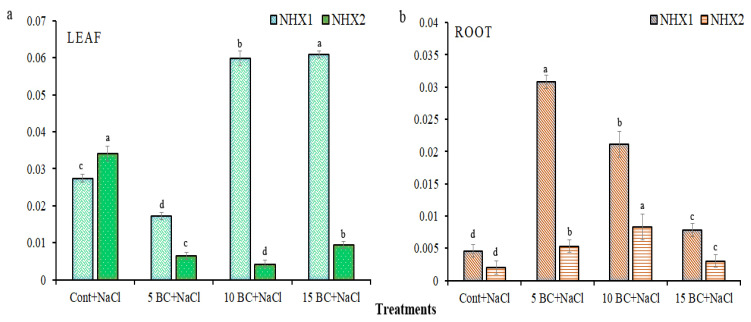
Quantitative real-time RT-PCR (qRT-PCR) analysis of *BoNHX* genes in the shoot and root tissues of Chinese cabbage grown under control and three difference concentrations of BC upon salinity. Expression levels of *BoNHX* genes in (**a**) leaf and (**b**) root samples of 90-day-old plants. The cycle threshold (Ct) values of Chinese cabbage leaf and root tissues were normalized to the constitutive gene EF-1α, and data were analyzed using the formula 2^–∆∆Ct^. Three biological replicates, each having technical triplicates (*n* = 12) of the leaf, and root samples, were used for qRT-PCR analysis. All values obtained from each trait were significantly different responses under various levels of BC and control (*p* < 0.05) based on a Tukey’s post-hoc test. Statistically significant analyses were performed separately for each trait between the control and BC treatments.

The phylogenetic tree was composed of three major clusters (MCs) (MC1 to MC3), which were further subdivided into one to two sub-clusters (SCs). MC1 clusters more NHX family proteins, with 14 NHXs, followed by MC2 and MC3, which cluster seven and four *NHXs*, respectively. *BoNHHX1* closely clusters with AtNHX1 and is distantly clustered with *AtNHX6* and *SlNHX2*. In MC2, *BoNHX2* closely clustered with *AtNHX2* and was distantly associated with other *NHXs*. The results clearly showed that the designed NHX proteins from this study aligned mostly with the NHX proteins of Arabidopsis, confirming the accuracy of our proteins. Both genes were analyzed in 90-day-old leaves and root tissues of Chinese cabbage grown under control and three different concentrations of BC with salinity stress. It is noteworthy that both genes showed expression in both the control and all BC treatments under salinity stress. Specifically, leaf tissue under 1.0 and 15 t/ha BC significantly upregulated the *BoNHX1* gene compared to the control and 5 t/ha BC. For instance, the leaf tissue showed a greater than four-fold upregulation in the *BoNHX1* compared to 5 t/ha BC and a greater than two-fold upregulation in the same tissue compared to the control (Figure 12a). Conversely, the leaf tissue under 5 t/ha BC had a decrease in its expression level compared to the control, and there was a significant difference in the expression level between the treatments.

However, there was no significant variation in its expression between 10 and 15 t/ha BC. The control significantly increased the expression level of *BoNHX2* by greater than threefold compared to all BC treatments. *BoNHX2* exhibits a higher upregulation in the leaf under 15 t/ha BC compared to the other two BC treatments; however, there are notable differences between 0.5 and 15 t/ha BC. Similar to leaf tissue, all BC treatments significantly increase the expression of the *BoNHX1* family gene in root tissue compared to the control (Figure 12b). However, the expression level in 5 t/ha BC was greater than twofold higher than in other BC treatments and control, and the expression pattern was significantly different. Like the *BoNHX1* gene, the *BoNHX2* gene also showed higher expression in the roots with all BC treatments compared to the control; however, it had a greater expression level in the 15 t/ha BC treatment than in the control and the other two BC treatments. Overall, the gene expression analysis showed that BC more strongly induced both genes than the control, and in both leaf and root tissues, the *BoNHX1* gene increased its expression level significantly more than the *BoNHX2* gene.

### 2.7. Principal Component Analysis (PCA) and Correlation Analysis Between Key Parameters

This study evaluated the relationships between morphological, physiological, and biochemical traits of Chinese cabbage grown under different BC concentrations in salinity stress conditions using heatmap and PCA analyses. The correlation analysis revealed significant relationships between soil properties, plant growth parameters, and nutrient content in Chinese cabbage under BC and salinity stress conditions. Plant growth, biomass, yield, and pigment traits, like LFW and TW (r = 0.97), as well as total chlorophyll (a + b) and SPAD values (r = 0.93), were strongly linked in the heatmap (Supplementary Figure S1). Soil Ca and Mg also displayed a high correlation (r = 0.88), suggesting a synergistic effect on nutrient uptake under salinity stress. We observed negative correlations (r = −0.88) between soil NH_4_^+^-N and NO_3_^−^-N (r = −0.88), suggesting that increased NH_4_^+^-N may suppress NO_3_^−^-N availability under saline conditions.

**Figure 13:**
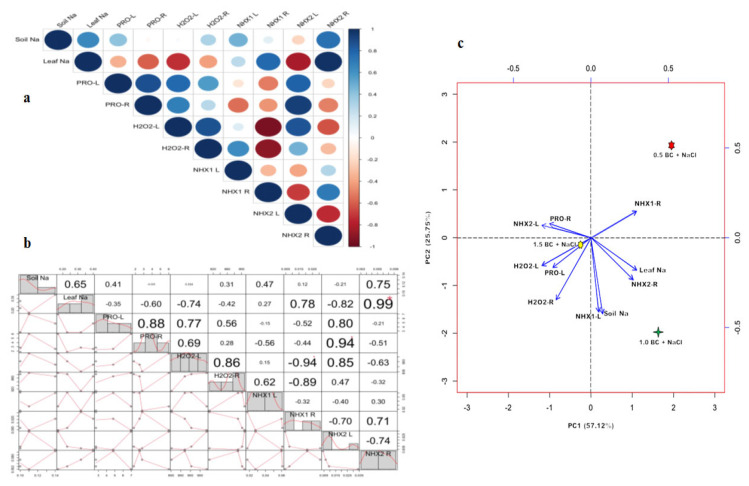
Pearson’s correlation and principal component analysis (PCA) analysis of biochemical and gene expression of Chinese cabbage plants exposed to salinity stress under control and three difference concentrations of BC. Brown and blue color indicate negative and positive correlation, respectively. (**a**) Cross correlation among biochemical and gene expression variables. (**b**) Pearson’s correlation matrix. (**c**) PCA analysis. (Top to bottom and left to right—soil Na; leaf Na; PRO-L; PRO-R; H_2_O_2_-L; H_2_O_2_-R; *BoNHX1 L; BoNHX1 R; BoNHX2 L; BoNHX2 R*).

The PCA plot provided insights into the effects of BC on stress-related traits, focusing on ion regulation and oxidative responses. The first two factors together explained 82.87% of the variation. PC1 (57.12%) showed links between Na levels (soil and leaf) and *NHX* gene expression in both leaves (*BoNHX1-L*) and roots (*BoNHX1-R, BoNHX2-R*). PC2 (25.75%) caught oxidative and osmoprotectant responses, and levels of proline and H_2_O_2_ in leaves and roots strongly correlated with *BoNHX2-L* (Figure 13a–c). Additionally, soil Na and leaf Na show a moderate positive correlation (r = 0.645), suggesting that soil Na levels influenced the leaf Na accumulation. In addition, gene regulation plays a crucial role in maintaining Na balance under salt stress. The evidence indicates a coordinated response to salinity, which involves proline accumulation, ROS regulation, and Na homeostasis. Overall, these analyses suggest that BC enhances nutrient availability, stress resilience, and ion balance, improving Chinese cabbage performance in saline conditions.

## 3. Discussion

Salinity-induced soil degradation and climate change severely impact global agriculture by reducing soil fertility and crop productivity. BC has shown promise as a sustainable solution, improving soil fertility, stabilizing nutrients, and reducing toxic elements [56,57]. BC has been shown to significantly enhance the relative abundance of Proteobacteria, one of the most functionally diverse and ecologically significant bacterial phyla in soil ecosystems. This group includes a wide range of beneficial microbial taxa involved in key biogeochemical processes, particularly the N cycle. In this study, BC-treated soils, particularly at 5 t/ha, exhibited higher pH, EC, OM, and nutrient availability than the control, creating a more favorable environment for plant growth (Figure 1). This aligns with previous reports of BC’s alkaline nature increasing soil pH [39,58] and improving EC to support nutrient uptake and ionic balance [59]. Consistent with Huang et al. [37], BC enhanced cation exchange capacity and the availability of K, Mg, and Ca. Elevated P levels across treatments suggest improved P solubility due to reduced fixation. BC also enhanced NH_4_^+^N and NO_3_^−^N retention, contributing to better nitrogen cycling [60], supported by Chen et al. [61], who reported that BC’s porous structure improves nutrient retention in saline soils. Additionally, BC improved soil water retention and lowered bulk density (BD), facilitating root growth under salinity stress findings that align with Hou et al. [62]. These soil improvements likely contributed to enhanced plant growth [63].

Numerous studies have examined the negative impact of salinity on photosynthesis, which is fundamental for plant development [64,65]. Our study revealed that photosynthetic pigments, such as chlorophyll and carotenoids, increased in all BC treatments, whereas control plants showed decreased pigment levels. This outcome aligns with findings in crops like beans, wheat, maize, cabbage, tomatoes, eggplant, and sorghum [35,36,66,67]. The observed rise might be because BC can increase N uptake, improve chloroplast structure, lower Na+ content, and raise chlorophyll synthesis and biomass growth [29,68]. Similarly, salt stress decreased LWC, but adding BC made plants hold on to more water, which is similar to what was found in bean, sorghum, and tomato plants, where BC increased water retention and toughness [32,67].

High saltiness affects plant growth, the balance of nutrients, and the production of ROS. This phenomenon causes oxidative stress and cell damage by affecting important cellular functions through biomolecular oxidation and lipid degradation [68]. Our results show that BC reduced stress caused by NaCl, significantly decreasing proline and H_2_O_2_ levels in both leaves and roots compared to the control. Researchers have reported similar beneficial effects of BC under abiotic stress conditions in wheat, cabbage, and beans [69,70]. Our study found that BC helps prevent cell damage caused by oxidative stress markers like O_2_^−^ and H_2_O_2_, probably because it makes plants more resilient to salt stress [71].

Previous studies have shown that salinity stress disrupts plant growth stages and impacts morphological, physiological, and biochemical processes, ultimately reducing crop yields [72,73]. We conducted this study to evaluate the impact of salinity stress on various growth parameters of Chinese cabbage, utilizing BC supplements as an alleviation strategy. Salinity stress alone significantly reduced growth, biomass, and yield traits such as LFW, RFW, LDW, RDW, LW, LL, RL, TNL, and TNR. It is possible that the lower growth rates seen in Chinese cabbage under salinity stress alone are caused by osmolytes building up, cells not dividing properly, and changes in metabolic enzyme activities and pathways [74,75]. However, BC application significantly improved all these growth, biomass, and yield traits under salinity stress compared to the control. This good effect on morphological traits is in line with what Akhtar et al. [29] found when they put BC on potatoes that were growing in salty conditions. They found that RL, root volume, and tuber yield all went up. In both soybean and common bean, adding BC increased SDW and RDW, which in turn increased total plant biomass [30]. These findings parallel our study, where BC treatment resulted in enhanced LDW and RDW in Chinese cabbage, indicating better nutrient acquisition and stress tolerance.

In addition to morphological improvements, our study also found that BC treatments increased the yield (TW) of Chinese cabbage under salinity stress. The results of our study are in agreement with those of Ekinci et al. [70], who reported that BC treatment in Chinese cabbage increased leaf area, as well as LFW, LDW, RFW, and RDW, ultimately boosting yield. In a related study, [76] found that BC enhanced both fruit yield and number in tomatoes under salinity stress, improving overall productivity. According to Duan et al. [77], adding BC to maize and wheat greatly increases overall yield. These yield enhancements across different crops highlight the role of BC in mitigating salinity stress, likely through improved nutrient availability, soil structure, and reduced Na^+^ toxicity.

The application of BC under salinity stress significantly enhanced macro- and micronutrient content in Chinese cabbage, consistent with previous findings [9,70]. BC-treated plants showed increased levels of key macronutrients such as P, K, Ca, and Mg, which are vital for physiological processes related to stress tolerance. For example, P content rose from 10.38% in the control to 17.34% at 15 t/ha, improving energy transfer and root development. In another study, the application of BC to saline soils was found to increase phosphorus availability [78]. Similarly, K levels increased moderately, aiding osmotic regulation, in line with rice studies that reported improved K uptake under BC treatment. Higher Ca and Mg concentrations observed in BC treatments contribute to cell wall stability and photosynthesis, both crucial under salinity.

Moreover, BC reduced Na uptake, helping alleviate ionic toxicity [79]. Enhanced availability of Fe and Mn was also observed, consistent with [57], supporting chlorophyll synthesis and enzyme function under stress. Zn and Cu levels increased with BC addition, though Cu declined slightly at higher BC rates, possibly due to competitive uptake or BC binding differences. Despite this, Zn availability was consistently improved, supporting enzyme activity and protein synthesis under salinity [78]. Overall, these nutrient enhancements contribute to BC’s role in mitigating salinity stress.

Reports suggest that *NHX* family genes may improve salt tolerance in plants by preserving cellular ion homeostasis. *NHX* family genes have been identified in numerous plant species; however, the *NHX* family genes have not yet been studied in Chinese cabbage. It is more important that no one has identified the role of salinity stress-responsive genes (including *NHX* family genes) under salinity stress supply with BC. This study identified two *NHX* family genes for Chinese cabbage, aligning with previous studies that identified 23 in wheat, 14 in soybean, 8 in tomato, 10 in pomegranate, 26 in pumpkin, and 8 in chickpea [51,52,53,80,81,82]. This suggests that Chinese cabbage exhibits a comparatively lower genetic diversity within this gene family. The *NHX1* gene is found on the tonoplast of Arabidopsis. It helps separate Na+ ions into vacuoles and makes the plant better able to handle salt stress. In our study, the *BoNHX1* gene was highly upregulated in the leaf tissue of Chinese cabbage grown under both 1.0 and 15 t/ha BC. As in our study, the *NHX1* gene was highly upregulated in the leaf tissues of wheat, soybean, pomegranate, pumpkin [51,52,80,81], and more under salinity stress. All BC treatments induced the *BoNHX2* gene in root tissue of Chinese cabbage under salinity stress in our study. In wheat, the expression level of *TaNXH2* was higher in roots under salinity stress and plays a vital role in conferring salinity stress tolerance [83]. Another study [84] found a highly upregulated *HvNHX2* gene in the root tissues of barley under salinity stress. These results clearly indicate that the *NHX* gene of plants (including Chinese cabbage) may enhance salinity stress tolerance. In addition, both genes identified from this study may lead to the sequestration of Na^+^ in the leaves and roots during salinity conditions. Therefore, overexpressing both genes in Chinese cages would aid in pinpointing its precise location. In addition, functional characterization (knockout) of these genes via genome editing tools would aid in identifying the exact role of both genes in Chinese cabbage. Genome editing tools have targeted several salinity stress-responsive genes in recent times. Therefore, these studies hold greater significance in this field of research, as they aim to enhance the production of Chinese cabbage while mitigating salinity stress.

## 4. Materials and Methods

### 4.1. Experimental Conditions

Seedlings of Chinese cabbage (Cv. Power Chungwang; 25 days old) were obtained from an NJ farm, Jeollabuk-do, Republic of Korea, and they were used for the entire experiment. The seedlings were removed from their containers and transplanted into a natural field under two different treatments: (1) salinity stress (200 mM NaCl) with the addition of three different concentrations of *Quercus acutissima* woodchip BC (e.g., 200 mM NaCl + 5 t/ha BC, 200 mM NaCl + 10 t/ha BC, and 200 mM NaCl + 15 t/ha BC) and (2) salinity stress (200 mM NaCl) without the addition of BC (hereafter referred to as the control). Woodchip BC was commercially obtained from Sanglim suppliers in the Jeollabuk-do, Republic of Korea. The BC had a cation exchange capacity of 24.7 cmol^+^/kg and was produced in a rotary kiln at 700 °C for 1 h, maintaining the oxygen concentration below 5% inside the pyrolysis chamber. The prepared BC sample has a neutral to slightly acidic pH of 6.55, a relatively low electrical conductivity (EC) of 0.34 dS/m, and a moisture content of 25.59%. The BC contained 71.57% organic matter, 1.24% nitrogen, 0.03% phosphoric acid, 0.16% potassium, 0.005% soluble kaolin, 0.004% boron, 0.001% iron, and 0.00002% molybdenum. The BC grain size at the time of application was below 2 cm, and the filling density varied based on application rate: 1.67 kg/m^3^ (5 t/ha), 3.33 kg/m^3^ (10 t/ha), and 5.00 kg/m^3^ (15 t/ha). BC was initially incorporated into the soil before transplanting the Chinese cabbage seedlings and thoroughly tilled into the soil to a depth of 30 cm to ensure even distribution. However, no BC was applied to the control. Next, we planted the Chinese cabbage seedlings separately in BC mixed soil and without BC mixed soil. The experiment was conducted from September to November 2023 at the National Institute of Horticultural and Herbal Science, RDA, Wanju-gun 55365, Jeollabuk-do, Republic of Korea (35.83 latitude, 127.03 longitude and altitude 56 m). The annual mean temperature was 12.5 °C, and the annual precipitation was 1326.8 mm. The soil used in this study was classified as SAMGAG (SgD3) and characterized as clay loam in texture, collected from the A horizon (surface layer). It was non-saline under natural conditions, with an EC of 0.198 dS/m and a pH of 5.16. The soil contained 1.28% OM, 16.91 mg/kg of available P_2_O_5_, and 0.199 cmolc/kg of exchangeable K^+^. The 200 mM NaCl concentration for this experiment was chosen based on a previous report by Duan et al. [77] to simulate salt stress conditions. Additionally, supplementary fertilizers were applied according to Korean standard recommendations for Chinese cabbage cultivation, including urea (0.3 t/ha), potassium chloride (0.23 t/ha), fused phosphate (1.0 t/ha), calcium (1.0 t/ha), and borax (0.15 t/ha). Each treatment was replicated twice in plots measuring (L × W; 40 m × 2 m), ensuring consistent conditions and minimizing edge effects, with adequate spacing to prevent cross-contamination. Irrigation was applied uniformly based on weather conditions, usually once a week. In addition, weeding and pest management practices were conducted across all plots.

### 4.2. Effects of BC on Soil Traits

Soil samples were collected from both control and all BC-treated plots (30 days’ post-application period) to a depth of 30 cm to assess the impact of BC application on soil quality. Soil samples were air-dried, ground, and passed through a 2 mm sieve prior to further analysis. The chemical and physical properties, such as pH, EC, OM, N, P, exchangeable cations, WC, BWC, and BD, of the soil were determined in control and all BC-supplemented soils under salinity stress following standard procedures by the National Institute of Agricultural Science and Technology, RDA, South Korea [85].

#### 4.2.1. pH and Electrical Conductivity (EC)

The soil pH and EC were measured using a soil and water (1:5) ratio. For example, 5 g of sieved soil was mixed with 25 mL of distilled water and stirred for 30 min. The pH of the suspension was then measured using an ORION Versa Star Meter (Thermo Fisher Scientific, MA, USA). EC was determined on the same soil suspension using a CM-30R EC meter (TODKK, Tokyo, Japan).

#### 4.2.2. Organic Matter (OM)

The analysis of soil OM (5 g soil) by the Tyurin method involves the oxidation of organic carbon with potassium dichromate. We then titrated the remaining dichromate against ferrous ammonium sulfate to determine the organic carbon content, which we then converted to OM percentage. Absorbance readings were taken using a UV-VIS NIR spectrophotometer (UV-3150, Shimadzu, Kyoto, Japan) to quantify the OM content.

#### 4.2.3. Exchangeable Cations

Exchangeable cations like K, Ca, Mg, and Na were taken from 5 g of soil that was mixed for 1 h with 50 mL of 1N ammonium acetate at a pH of 7.0, and then the mixture was filtered. The concentrations of all exchangeable cations were determined using an inductively coupled plasma (ICP) spectrometer (Integra, GBC Scientific Equipment, Melbourne, Australia).

#### 4.2.4. Nitrogen (N) and Phosphorus (P) Content

Soil N content, specifically ammonium (NH_4_^+^N) and nitrate (NO_3_^−^N), was determined after reduction of NO_3_^−^ to NH_4_^+^ using the Kjeldahl method. A 5 g soil sample was shaken with 50 mL of 2 M KCl for 1 h to extract the available N forms. The concentration of available P was determined using the Lancaster method. For example, approximately 5 g of soil sample were shaken with 50 mL of Lancaster reagent (0.03 M ammonium fluoride and 0.025 M hydrochloric acid) for 30 min. The P content in the filtrate was determined at a wavelength of 880 nm using a UV-VIS-NIR spectrophotometer (UV-3150, Shimadzu, Kyoto, Japan).

#### 4.2.5. Water Holding Capacity

The impact of BC treatment on soil water-holding capacity was evaluated using 5 g of soil by analyzing WC, BWC, and BD of soil samples. WC was determined by the weight difference of soil samples before and after oven drying at 105 °C, calculated as WC (%) = (weight of wet soil − weight of dry soil/weight of dry soil) × 100. BWC was calculated by BWC (%) = WC (%) × BD (g/mL), and BD was obtained by using the formula BD (g/mL) = dry mass of soil (g)/volume of soil core (mL), respectively.

### 4.3. Effects of BC on Biochemical Traits of Chinese Cabbage

All the biochemical traits were collected from 90-day-old Chinese cabbage grown under control and salinity stress with three different concentrations of BC.

#### 4.3.1. Photosynthetic Pigment Analysis

The chlorophyll a, b, and a + b and carotenoid contents were determined in the 90-day-old leaves of Chinese cabbage by Lichtenthaler and Buschmann [86]. Around 100 mg of leaves were finely ground with 1 mL of 98% methanol and incubated at 4 °C for 24 h in complete darkness. The same 98% methanol solution was used as the blank. The chlorophyll content was measured at 652.4 nm and 665.2 nm, and the carotenoid content was measured at 480 nm using a microplate reader (EPOCH2, BioTek Instruments Inc., Winooski, VT, USA). The SPAD values were measured using an SPAD-5We and an SPAD-502 plus chlorophyll meter (Konica Minolta, Tokyo, Japan) to take measurements between 9:00 and 10:00 am with three biological replicates for both control and BC treatment groups, providing a non-destructive estimation of chlorophyll content.

#### 4.3.2. Leaf Water Content (LWC)

LWC was analyzed in 90-day-old Chinese cabbage leaves grown under control and all BC concentrations following salinity treatment. About 1000 mg of leaves were placed in an oven until the weight remained constant at 105 °C for 72 h, then the LWC was calculated by the following formula: LWC = LFW − LDW/LFW × 100.

#### 4.3.3. Proline and Hydrogen Peroxide (H_2_O_2_) Analysis

Approximately 100 mg of leaf and root samples from both control and all BC-treated plants were finely ground to determine free proline content by Bates [87] and H_2_O_2_ content by Del Pozo and Lam [88]. The absorbance for proline was measured at 520 nm and for H_2_O_2_ at 390 nm using a microplate reader (EPOCH2, BioTek Instruments Inc., Winooski, VT, USA). The final concentrations of proline and H_2_O_2_ were calculated by comparing the absorbance values with standard curves prepared using L-proline and H_2_O_2_ standards, respectively.

### 4.4. In Situ Analysis of Superoxide Anion (O_2_^−^) and H_2_O_2_

The histochemical detection of O_2_^−^ and H_2_O_2_ in the leaves of Chinese cabbage was performed using the chromogenic substrates DAB and NBT, following the procedure described in our previous study by Rathinapriya et al. [73]. Briefly, an innermost leaf was excised and immersed in 0.1% DAB (for H_2_O_2_) and 0.1% NBT (for O_2_^−^) solutions in 10 mM potassium phosphate buffer for 3–5 h at room temperature. The samples were then destained with an acetic acid-ethanol solution (1:3 *v*/*v*) to remove chlorophyll and then photographed. The intensity and distribution of the blue (O_2_^−^) and brown (H_2_O_2_) formazan precipitates were used to qualitatively assess ROS levels in the different treatments.

### 4.5. Effects of BC on Phenotypic Traits of Chinese Cabbage

Phenotypic traits such as LFW, LDW, LL, RL, TNL, and TNR were collected from 90-day-old Chinese cabbage plants grown under control and three different BC-supplemented soils under salinity stress. For measurement of LL and RL, a stainless steel ruler was used. Before measuring the RL, roots were carefully removed with plants from the soil, rinsing them with water to avoid damaging the root system, blotting them dry with tissue paper, and then measuring the length with a ruler. Similarly, L was measured from the base of the Chinese cabbage core to the tip of the tallest leaf. To determine the LDW, plant samples were dried in an oven at 70 °C until a constant weight was achieved, then allowed to cool to RT before weighing. At last, yield traits such as NLPH, ML, MW, and TW were evaluated after 90-day-old plants were grown in control and combinations of three concentrations of BC upon salinity stress. For each treatment, data were collected from three plants, and the mean value was calculated (*n* = 12).

### 4.6. Analysis of Essential Macro- and Micronutrient Contents in Leaf Tissue of Chinese Cabbage

Eleven essential macro- and micronutrients, such as K, Ca, Mg, Na, Fe, Cu, P, Zn, Mn, T-N, and T-C, were analyzed in 90-day-old leaves of Chinese cabbage grown under control and three different BC-supplemented soils under salinity stress. The collected leaf samples were oven-dried for 5 days at 70 °C, and for each macro- and micronutrient content analysis, 500 mg of leaf samples were used, following the standard protocol outlined by the NLAST, 2000 [85]. The T-N and T-C contents were quantified using an elemental analyzer (Primacs SNC-100, Skalar, Breda, Netherlands). The P content was determined through the ammonium metavanadate method at a wavelength of 420 nm using a UV-VIS-NIR spectrophotometer (Bltec, AA3 UV-3150, Shimadzu, Kyoto, Japan). K, Ca, Mg, and Na were extracted from the samples using 1N ammonium acetate at pH 7.0 and amounts were determined using an ICP spectrometer (Integra, GBC Scientific Equipment, Melbourne, Australia).

The micronutrients Fe, Mn, Cu, and Zn were determined by wet digestion of 500 mg of the dry powdered sample in a nitric acid and perchloric acid (85:15, *v*/*v*) mixture. The filtrate was used to detect and quantify the levels of micronutrients by measuring the emission spectra of ionized atoms in the ICP spectrometer (Integra, GBC, Melbourne, Australia).

### 4.7. Molecular Studies

#### 4.7.1. Collection of Nucleotide and Protein Sequences of *NHX* Family Genes for Chinese Cabbage

The protein sequences of *NHX* family genes from Arabidopsis were compared to the cabbage genome assembly (*Brassica oleracea* capitata v1.0 assembly) on the Phytozome 8 website, and two *BoNHX* family genes (*BoNHX1* and *BoNHX2*) were identified in the cabbage genome assembly. Aside from these two genes, no other *NHX* family members are present in the *Brassica oleracea* genome because the genome is incompletely annotated. Selection was based on query cover (80–100%), except value = 0, presence of start codon “ATG,” scaffolds, and sequencing range. Based on these components, we downloaded the genomic, CDS, and protein sequences of *BoNHX* family genes. The collected *BoNHX* family protein sequences were validated by the UniProt database, confirming them as *BoNHX* family transport proteins, and each gene name was assigned to Chinese cabbage based on sequence similarity to Arabidopsis.

#### 4.7.2. Analysis of Protein Features of BoNHX and Gene Structure of *BoNHX*

The number of transmembrane domains (TMDs) of each *BoNHX* gene was predicted by TMHMM server v.2.0, an online-enabled tool (http://www.cbs.dtu.dk/services/TMHMM/, 25 January 2024). Physicochemical properties such as molecular weight (MW) and isoelectric point (pI) of each BoNHX protein were determined by the ProtParam tool on the ExPASy server (http://web.expasy.org/protparam/, 25 January 2024). The subcellular localization of each BoNHX protein was predicted by the WoLF PSORT server (https://www.genscript.com/wolf-psort.html/, 25 January 2024). The full-length genomic sequences of *BoNHX* genes were used to determine the intron/exon distribution by the gene structure display server program (GSDS, http://gsds.cbi.pku.edu.cn/, 25 January 2024). The molecular evolutionary genetics analysis v6 software tool constructed a phylogenetic tree using the protein sequences of Chinese cabbage’s *BoNHX* family transporters and those of other plants. We maintained the bootstrap value of 1000 replicates to construct the phylogenetic tree.

#### 4.7.3. RNA Isolation and cDNA Synthesis

For RNA isolation, leaves and root samples were collected from 90-day-old Chinese cabbage grown under control and salinity stress with three different BC concentrations and immediately stored at −80 °C until further use. The RNeasy Plant Mini Kit (Qiagen, Hilden, Germany) was used to get total RNA from three frozen samples of leaves and root tissue for each treatment. The kit’s instructions were followed exactly. The purity and concentration of the isolated RNA were assessed using a NanoDrop spectrophotometer (ND-2000, Thermo Scientific, Waltham, MA, USA). cDNA synthesis was performed in triplicate from 500 ng of total RNA per sample using the QuantiTect Reverse Transcription Kit (Qiagen, Hilden, Germany). The three biological replicates were included for each sample in both RNA isolation and cDNA synthesis.

#### 4.7.4. Quantitative Real-Time RT-PCR (qRT-PCR)

The expression patterns of *BoNHX1* and *BoNHX2* were examined in the leaves and root tissues of Chinese cabbage grown under control and salinity stress with the application of three different concentrations of BC. For qRT-PCR analysis, the reactions were performed in a 10 μL volume containing 5 μL of iQTM SYBR^®^ Green Supermix (Bio-Rad Laboratories, Hercules, CA, USA), 1 μL of forward: reverse primer mix (500 nM each), and 4 μL of cDNA diluted to a 1:50 ratio. The cycling conditions were as follows: initial enzyme activation at 95 °C for 30 s, 34 cycles of denaturation at 95 °C for 5 s, and annealing and extension at 60 °C for 5 s. A melting curve analysis was conducted by gradually increasing the temperature from 65 to 95 °C in 0.5 °C increments, with continuous signal capture to ensure the specificity of the amplification. Cycle threshold (Ct) values for all samples were analyzed using the 2^–ΔΔ^ Ct method [89]. The Ct values were normalized against the expression of the constitutive gene *EF-1α*, which was chosen based on its stability in *Brassica* under salt conditions by previous studies [90]. Three biological replicates, each with three technical replicates, were used to ensure the accuracy and reliability of the qRT-PCR analysis.

### 4.8. Statistical Analysis

All experiments were conducted using a completely randomized design, ensuring a minimum of three replicates for each treatment. Data from three randomly selected plants in each row (n (3 × 4) = 12) were expressed as mean ± standard deviation (SD). Statistical significance among mean values was determined using one-way analysis of variance (ANOVA), followed by Tukey’s post-hoc test. The analyses were performed using SAS Enterprise Guide 7.1 (SAS Institute Inc., Cary, NC, USA) at the *p* < 0.05 significance level. Pearson’s correlation and principal component analysis (PCA) were performed using the “FactoMineR” and “Factoextra” packages in R software version 4.2.2.

## 5. Conclusions

This study provides novel insights into the potential of BC as an effective soil amendment for enhancing salinity tolerance in Chinese cabbage. Our results demonstrate that BC significantly improves soil properties, nutrient availability, and water-holding capacity, contributing to a more favorable environment for plant growth under saline conditions. Based on our observations, the application of 5 t/ha BC enhanced various physiological and biochemical traits of Chinese cabbage under salinity stress compared to the other treatments. However, the macro- and micronutrient contents were significantly lower at 5 t/ha compared to higher BC application rates. We assume that plants at the 5 t/ha treatment absorbed a sufficient amount of nutrients to support their growth under salinity stress, which contributed to the significant improvement in physiological traits. Therefore, the application of 5 t/ha BC may be optimal for enhancing the growth and yield of Chinese cabbage under salinity stress conditions. BC increases Na^+^ sequestration by increasing *BoNHX* gene expression in both leaf and root tissues. This process supports ion homeostasis, which is important for salt tolerance. Furthermore, BC treatments lowered signs of oxidative stress, like proline and H_2_O_2_, indicating an antioxidant response that reduces harm from salinity. Heatmap and PCA analyses demonstrate the intricate interactions between soil nutrients, physiological traits, and biochemical stress responses. Overall, this study establishes BC as a promising eco-friendly amendment to improve crop productivity in saline-prone soils, supporting sustainable agricultural practices and food security in salt-affected regions.

## Data Availability

The original contributions presented in this study are included in the article. Further inquiries can be directed to the corresponding author.

## Supplementary Materials

The following supporting information can be downloaded at https://www.mdpi.com/article/10.3390/plants14172743/s1. Figure S1: Heatmap of Pearson’s correlation analysis of BC treatment on the soil properties, plant morphological, physiological, and biochemical parameters of Chinese cabbage exposed to salinity stress; Table S1: Details of NHX family transporters of Chinese cabbage.

## Abbreviations

DAB	3,3-diaminobenzidine
BC	biochar
BD	bulk density
BWC	bulk water content
Ca	calcium
C	carbon
CAR	carotenoid
CHL	chlorophyll
EC	electrical conductivity
H_2_O_2_	hydrogen peroxide
Fe	iron
LL	leaf length
LWC	leaf water content
LDW	leaves dry weight
LFW	leaves fresh weight
Mg	magnesium
Mn	manganese
ML	midrib length
MW	midrib width
N	nitrogen
NBT	nitrotetrazolium blue chloride
NLPH	number of leaves per head
OM	organic matter
P	phosphorus
K	potassium
PCA	principal component analysis
PRO-L	leaf proline
PRO-R	root proline
ROS	reactive oxygen species
RT	room temperature
RL	root length
NaCl	sodium chloride
Na+	sodium ions
O_2_^−^	superoxide anion
T-C	total carbon
T-N	total nitrogen
TNL	total number of leaves
TNR	total number of roots per plant
TW	total weight
WC	water content
Zn	zinc

## References

[1] United States Department of Agriculture (USDA) (2023). Food Data Central: Chinese Cabbage, Raw. https://fdc.nal.usda.gov/.

[2] Stefan I.M.A., Ona A.D. (2020). Cabbage (Brassica oleracea L.): Overview of the Health Benefits and Therapeutical Uses. https://odontoanamaria.com/artigos/repolho01.pdf.

[3] Sarkar D., Rakshit A., Parewa H.P., Danish S., Alfarraj S., Datta R. (2022). Bio-priming with compatible rhizospheric microbes enhances growth and micronutrient uptake of red cabbage. Land.

[4] Kim J., Lee J., Jang Y., Lee S., Lee W.M., Wi S., Yoon H.I. (2024). Elucidating Genetic Mechanisms of Summer Stress Tolerance in Chinese Cabbage through GWAS and Phenotypic Analysis. Agronomy.

[5] Hongu N., Kim A.S., Suzuki A., Wilson H., Tsui K.C., Park S. (2017). Korean kimchi: Promoting healthy meals through cultural tradition. J. Ethn. Foods.

[6] Kim S., Rho H.Y., Kim S. (2022). The Effects of Climate Change on Heading Type Chinese Cabbage (*Brassica rapa* L. ssp. Pekinensis) Economic Production in South Korea. Agronomy.

[7] Kayum M.A., Kim H.T., Nath U.K., Park J.I., Kho K.H., Cho Y.G., Nou I.S. (2016). Research on biotic and abiotic stress related genes exploration and prediction in *Brassica rapa* and *B. oleracea*: A review. Plant Breed. Biotechnol..

[8] Jabeen A., Mir J.I., Malik G., Yasmeen S., Ganie S.A., Rasool R., Hakeem K.R. (2024). Biotechnological interventions of improvement in cabbage (*Brassica oleracea* var. *capitata* L.). Sci. Hortic..

[9] Li X., Ayub M.A., Fox J.P., Shen S., Rossi L. (2024). Nutrient uptake, growth, and physiology of Chinese cabbage (*Brassica rapa* L. ssp. *pekinensis*) varieties under NaCl stress. Soil Environ..

[10] Sande T.J., Tindwa H.J., Alovisi A.M.T., Shitindi M.J., Semoka J.M. (2024). Enhancing sustainable crop production through integrated nutrient management: A focus on vermicompost, bio-enriched rock phosphate, and inorganic fertilisers—A systematic review. Front. Agron..

[11] Al-Shammary A.A.G., Al-Shihmani L.S.S., Fernández-Gálvez J., Caballero-Calvo A. (2024). Optimizing sustainable agriculture: A comprehensive review of agronomic practices and their impacts on soil attributes. J. Environ. Manag..

[12] Yasmeen A.R., Maharajan T., Rameshkumar R., Sindhamani S., Banumathi B., Prabakaran M., Atchaya S., Rathinapriya P. (2025). Role of Seaweeds for Improving Soil Fertility and Crop Development to Address Global Food Insecurity. Crops.

[13] Machado R.M.A., Serralheiro R.P. (2017). Soil salinity: Effect on vegetable crop growth. Management practices to prevent and mitigate soil salinization. Horticulturae.

[14] Singh A. (2022). Soil salinity: A global threat to sustainable development. Soil Use Manag..

[15] Mishra A.K., Das R., George Kerry R., Biswal B., Sinha T., Sharma S., Kumar M. (2023). Promising management strategies to improve crop sustainability and to amend soil salinity. Front. Environ. Sci..

[16] Okorogbona A.O.M., Managa L.R., Adebola P.O., Ngobeni H.M., Khosa T.B. (2015). Salinity and crop productivity. Sustain. Agric. Rev..

[17] Safdar H., Amin A., Shafiq Y., Ali A., Yasin R., Shoukat A., Sarwar M.I. (2019). A review: Impact of salinity on plant growth. Nat. Sci..

[18] Nielsen S., Joseph S., Ye J., Chia C., Munroe P., van Zwieten L., Thomas T. (2018). Crop-season and residual effects of sequentially applied mineral enhanced biochar and N fertiliser on crop yield, soil chemistry and microbial communities. Agric. Ecosyst. Environ..

[19] Hossain M.Z., Bahar M.M., Sarkar B., Donne S.W., Ok Y.S., Palansooriya K.N., Kirkham M.B., Chowdhury S., Bolan N. (2020). Biochar and its importance on nutrient dynamics in soil and plant. Biochar.

[20] Singh H., Northup B.K., Rice C.W., Prasad P.V. (2022). Biochar applications influence soil physical and chemical properties, microbial diversity, and crop productivity: A meta-analysis. Biochar.

[21] Gao S., DeLuca T.H. (2016). Influence of biochar on soil nutrient transformations, nutrient leaching, and crop yield. Adv. Plants Agric. Res..

[22] De Vasconcelos A.C.F. (2020). Biochar effects on amelioration of adverse salinity effects in soils. Applications of Biochar for Environmental Safety.

[23] Xiao Q., Zhu L.X., Zhang H.P., Li X.Y., Shen Y.F., Li S.Q. (2016). Soil amendment with biochar increases maize yields in a semi-arid region by improving soil quality and root growth. Crop Pasture Sci..

[24] Adekiya A.O., Agbede T.M., Olayanju A., Ejue W.S., Adekanye T.A., Adenusi T.T., Ayeni J.F. (2020). Effect of biochar on soil properties, soil loss, and cocoyam yield on a tropical sandy loam Alfisol. Sci. World J..

[25] Kumari K., Khalid Z., Alam S.N., Sweta, Singh B., Guldhe A., Shahi D.K., Bauddh K. (2020). Biochar amendment in agricultural soil for mitigation of abiotic stress. Ecological and Practical Applications for Sustainable Agriculture.

[26] Karimi A., Moezzi A., Chorom M., Enayatizamir N. (2020). Application of biochar changed the status of nutrients and biological activity in a calcareous soil. J. Soil Sci. Plant Nutr..

[27] Yao Q., Liu J., Yu Z., Li Y., Jin J., Liu X., Wang G. (2017). Three years of biochar amendment alters soil physiochemical properties and fungal community composition in a black soil of Northeast China. Soil Biol. Biochem..

[28] Alkharabsheh H.M., Seleiman M.F., Shami A., Al-Gheraibah H.M., Ammar K.A., Alkahtani J., Battaglia M.L. (2021). Biochar and its impact on soil fertility, nutrient leaching, and crop productivity: A review. Agronomy.

[29] Akhtar S.S., Andersen M.N., Liu F. (2015). Biochar mitigates salinity stress in potato. J. Agron. Crop Sci..

[30] Farhangi-Abriz S., Torabian S. (2018). Biochar improved nodulation and nitrogen metabolism of soybean under salt stress. Symbiosis.

[31] She D., Sun Y., Liang Z., Jiang S., Zhang L. (2018). Biochar increased photosynthesis, transpiration rate, yield, and fruit number in tomato under salinity stress. J. Plant Growth Regul..

[32] Ibrahim M., Abdel-Fattah M., Taha M. (2020). Effects of biochar on plant growth and yield under salinity stress in sorghum. Environ. Exp. Bot..

[33] Nikpour-Rashidabad R., Baghizadeh A., Gholamin R. (2019). The effect of biochar on physiological and anatomical characteristics of mung bean roots under salt stress. Arch. Biol. Sci..

[34] Soothar M.K., Mounkaila Hamani A.K., Kumar Sootahar M., Sun J., Yang G., Bhatti S.M., Traore A. (2021). Assessment of Acidic Biochar on the Growth, Physiology and Nutrients Uptake of Maize (*Zea mays* L.) Seedlings under Salinity Stress. Sustainability.

[35] Parkash V., Singh S. (2020). Potential of biochar application to mitigate salinity stress in eggplant. HortScience.

[36] Chen G., Wu F., Li X., Luo C. (2023). Biochar application enhances growth and yield of cabbage under salinity stress. Agron. J..

[37] Huang M., Yang L., Qin F., Jiang L., Zou Y. (2019). Effects of biochar on growth parameters and nutrient uptake in wheat under salinity stress. Soil Use Manag..

[38] Kanwal S., Ilyas N., Shabir S., Mahmood T., Akhtar N., Hussain T. (2018). Biochar application to mitigate negative effects of salinity stress on wheat. J. Plant Nutr..

[39] Yang A., Akhtar S.S., Li L., Fu Q., Li Q. (2020). Biochar mitigates combined salinity and drought stress in quinoa. Agronomy.

[40] Zhao S., Zhang Q., Liu M., Zhou H., Ma C., Wang P. (2021). Regulation of plant responses to salt stress. Int. J. Mol. Sci..

[41] Balasubramaniam T., Shen G., Esmaeili N., Zhang H. (2023). Plants’ response mechanisms to salinity stress. Plants.

[42] Ketehouli T., Idrice Carther K.F., Noman M., Wang F.W., Li X.W., Li H.Y. (2019). Adaptation of plants to salt stress: Characterization of Na+ and K+ transporters and role of *CBL* gene family in regulating salt stress response. Agronomy.

[43] Ji H., Pardo J.M., Batelli G., Van Oosten M.J., Bressan R.A., Li X. (2013). The salt overly sensitive (*SOS*) pathway: Established and emerging roles. Mol. Plant.

[44] Venkataraman G., Shabala S., Véry A.A., Hariharan G.N., Somasundaram S., Pulipati S., Chen Z.H. (2021). To exclude or to accumulate? Revealing the role of the sodium HKT1; 5 transporters in plant adaptive responses to varying soil salinity. Plant Physiol. Biochem..

[45] Sofo A., Scopa A., Nuzzaci M., Vitti A. (2015). Ascorbate peroxidase and catalase activities and their genetic regulation in plants subjected to drought and salinity stresses. Int. J. Mol. Sci..

[46] Hasanuzzaman M., Raihan M.R.H., Masud A.A.C., Rahman K., Nowroz F., Rahman M., Fujita M. (2021). Regulation of reactive oxygen species and antioxidant defense in plants under salinity. Int. J. Mol. Sci..

[47] Jiang X., Leidi E.O., Pardo J.M. (2010). How do vacuolar *NHX* exchangers function in plant salt tolerance?. Plant Signal. Behav..

[48] Akram U., Song Y., Liang C., Abid M.A., Askari M., Myat A.A., Meng Z. (2020). Genome-wide characterization and expression analysis of NHX gene family under salinity stress in *Gossypium barbadense* and its comparison with *Gossypium hirsutum*. Genes.

[49] Liu H., Wang K., Mei Q., Wang X., Yang J., Ma F., Mao K. (2023). Genome-wide analysis of the Actinidia chinensis *NHX* family and characterization of the roles of *AcNHX3* and *AcNHX7* in regulating salt tolerance in Arabidopsis. Environ. Exp. Bot..

[50] Ratner A., Jacoby B. (1976). Effect of K+, its counter anion, and pH on sodium efflux from barley root tips. J. Exp. Bot..

[51] Sharma P., Mishra S., Pandey B., Singh G. (2023). Genome-wide identification and expression analysis of the NHX gene family under salt stress in wheat (*Triticum aestivum* L). Front. Plant Sci..

[52] Joshi S., Kaur K., Khare T., Srivastava A.K., Suprasanna P., Kumar V. (2021). Genome-wide identification, characterization and transcriptional profiling of NHX-type (Na+/H+) antiporters under salinity stress in soybean. 3 Biotech.

[53] Cavusoglu E., Sari U., Tiryaki I. (2023). Genome-wide identification and expression analysis of Na+/H+ antiporter (*NHX*) genes in tomato under salt stress. Plant Direct.

[54] Wu G.Q., Wang J.L., Li S.J. (2019). Genome-wide identification of Na+/H+ antiporter (*NHX*) genes in sugar beet (*Beta vulgaris* L.) and their regulated expression under salt stress. Genes.

[55] Liu S., Liu Y., Yang X., Tong C., Edwards D., Parkin I.A., Paterson A.H. (2014). The *Brassica oleracea* genome reveals the asymmetrical evolution of polyploid genomes. Nat. Commun..

[56] Bian X., Ren Z., Zeng L., Zhao F., Yao Y., Li X. (2024). Effects of biochar on the compressibility of soil with high water content. J. Clean. Prod..

[57] Igalavithana A.D., Mandal S., Niazi N.K., Vithanage M., Parikh S.J., Mukome F.N., Ok Y.S. (2017). Advances and future directions of biochar characterization methods and applications. Crit. Rev. Environ. Sci. Technol..

[58] He K., Xu Y., He G., Zhao X., Wang C., Li S., Hu R. (2023). Combined application of acidic biochar and fertilizer synergistically enhances Miscanthus productivity in coastal saline-alkaline soil. Sci. Total Environ..

[59] Biederman L.A., Harpole W.S. (2013). Biochar and its effects on plant productivity and nutrient cycling: A meta-analysis. GCB Bioenergy.

[60] Harris E. (2011). Impact of Biochar Amendment on Nutrient Retention by Riparian Soils. Ph.D. Thesis.

[61] Chen X., Yang S.H., Jiang Z.W., Ding J., Sun X. (2021). Biochar as a tool to reduce environmental impacts of nitrogen loss in water-saving irrigation paddy field. J. Clean. Prod..

[62] Hou J., Yi G., Hao Y., Li L., Shen L., Zhang Q. (2024). The effect of combined application of biochar and phosphate fertilizers on phosphorus transformation in saline-alkali soil and its microbiological mechanism. Sci. Total Environ..

[63] Kang M.W., Yibeltal M., Kim Y.H., Oh S.J., Lee J.C., Kwon E.E., Lee S.S. (2022). Enhancement of soil physical properties and soil water retention with biochar-based soil amendments. Sci. Total Environ..

[64] Xu C.Y., Hosseini-Bai S., Hao Y., Rachaputi R.C.N., Wang H., Xu Z., Wallace H. (2015). Effect of biochar amendment on yield and photosynthesis of peanut on two types of soils. Environ. Sci. Pollut. Res..

[65] Abbas G., Amjad M., Saqib M., Murtaza B., Naeem M.A., Shabbir A., Murtaza G. (2021). Soil sodicity is more detrimental than salinity for quinoa (*Chenopodium quinoa* Willd.): A multivariate comparison of physiological, biochemical and nutritional quality attributes. J. Agron. Crop Sci..

[66] Akhtar S.S., Andersen M.N., Naveed M., Zahir Z.A., Liu F. (2015). Interactive effect of biochar and plant growth promoting bacterial endophytes on ameliorating salinity stress in maize. Funct. Plant Biol..

[67] Kul R., Arjumend T., Ekinci M., Yildirim E., Turan M., Argin S. (2021). Biochar as an organic soil conditioner for mitigating salinity stress in tomato. Soil Sci. Plant Nutr..

[68] Ullah I., Ali N., Durrani S., Shabaz M.A., Hafeez A., Ishfaq M., Fayyaz M.R., Rehman A., Waheed A. (2018). Effect of different nitrogen levels on growth, yield and yield contributing attributes of wheat. Int. J. Sci. Eng. Res..

[69] Parihar P., Singh S., Singh R., Singh V.P., Prasad M.S. (2015). Effect of salinity stress on plants and its tolerance strategies: A review. Environ. Sci. Pollut. Res..

[70] Ekinci M., Turan M., Yildirim E. (2022). Biochar mitigates salt stress by regulating nutrient uptake and antioxidant activity, alleviating the oxidative stress and abscisic acid content in cabbage seedlings. Turk. J. Agric. For..

[71] Hassan M.U., Aamer M., Chattha M.U., Ullah M.A., Sulaman S., Nawaz M., Zhiqiang W., Yanqin M., Guoqin H. (2017). The role of potassium in plants under drought stress: A mini review. J. Basic Appl. Sci..

[72] Hualpa-Ramirez E., Carrasco-Lozano E.C., Madrid-Espinoza J., Tejos R., Ruiz-Lara S., Stange C., Norambuena L. (2024). Stress salinity in plants: New strategies to cope with in the foreseeable scenario. Plant Physiol. Biochem..

[73] Rathinapriya P., Pandian S., Rakkammal K., Balasangeetha M., Alexpandi R., Satish L., Ramesh M. (2020). The protective effects of polyamines on salinity stress tolerance in foxtail millet (*Setaria italica* L.), an important C4 model crop. Physiol. Mol. Biol. Plants.

[74] Qiu N., Liu Q., Li J., Zhang Y., Wang F., Gao J. (2017). Physiological and transcriptomic responses of Chinese cabbage (*Brassica rapa* L. ssp. Pekinensis) to salt stress. Int. J. Mol. Sci..

[75] Pavlović I., Mlinarić S., Tarkowská D., Oklestkova J., Novák O., Lepeduš H., Salopek-Sondi B. (2019). Early Brassica crops responses to salinity stress: A comparative analysis between Chinese cabbage, white cabbage, and kale. Front. Plant Sci..

[76] Ud Din M.M., Khan M.I., Azam M., Ali M.H., Qadri R., Naveed M., Nasir A. (2023). Effect of biochar and compost addition on mitigating salinity stress and improving fruit quality of tomato. Agronomy.

[77] Duan S., Al-Huqail A.A., Alsudays I.M., Younas M., Aslam A., Shahzad A.N., Hong Yong J.W. (2024). Effects of biochar types on seed germination, growth, chlorophyll contents, grain yield, sodium, and potassium uptake by wheat (*Triticum aestivum* L.) under salt stress. BMC Plant Biol..

[78] Saifullah, Dahlawi S., Naeem A., Rengel Z., Naidu R. (2018). Biochar application for the remediation of salt-affected soils: Challenges and opportunities. Sci. Total Environ..

[79] Gao Z.W., Ding J., Ali B., Nawaz M., Hassan M.U., Ali A., Sabagh A.E. (2024). Putting Biochar in Action: A Black Gold for Efficient Mitigation of Salinity Stress in Plants. ACS Omega.

[80] Dong J., Liu C., Wang Y., Zhao Y., Ge D., Yuan Z. (2021). Genome-wide identification of the NHX gene family in *Punica granatum* L. and their expressional patterns under salt stress. Agronomy.

[81] Shen C., Yuan J., Li X., Chen R., Li D., Wang F., Li X. (2023). Genome-wide identification of NHX (Na+/H+ antiporter) gene family in *Cucurbita* L. and functional analysis of CmoNHX1 under salt stress. Front. Plant Sci..

[82] Parveen K., Saddique M.A.B., Rehman S.U., Ali Z., Aziz I., Shamsi I.H., Muneer M.A. (2023). Identification and characterization of salt stress-responsive *NHX* gene family in chickpea. Plant Stress.

[83] Yarra R. (2019). The wheat *NHX* gene family: Potential role in improving salinity stress tolerance of plants. Plant Gene.

[84] Kargar F., Niazi A., Fakhrfeshani M., Malekzadeh K. (2019). Expression analysis of genes encoding NHX2 antiporter and subunit A of vacuolar H+-ATPase pump in salt-resistant and salt-sensitive barley (*Hordeum vulgare* L.) cultivars under salt stress. Russ. J. Plant Physiol..

[85] NLAST (2000). Soil and Plant Analysis Method.

[86] Lichtenthaler H.K., Buschmann C. (2001). Chlorophylls and carotenoids: Measurement and characterization by UV-VIS spectroscopy. Curr. Proto. Food Anal. Chem..

[87] Bates L.S., Waldren R.P., Teare I.D. (1973). Rapid determination of free proline for water-stress studies. Plant Soil.

[88] Del Pozo O., Lam E. (1998). Caspases and programmed cell death in the hypersensitive response of plants to pathogens. Curr. Biol..

[89] Rao X., Huang X., Zhou Z., Lin X. (2013). An improvement of the 2ˆ(–delta delta CT) method for quantitative real-time polymerase chain reaction data analysis. Biostat. Bioinform. Biomath..

[90] Sheng X.G., Zhao Z.Q., Yu H.F., Wang J.S., Zheng C.F., Gu H.H. (2016). In-depth analysis of internal control genes for quantitative real-time PCR in *Brassica oleracea* var. botrytis. Genet. Mol. Res..

