# Supplementary material for: Biochar Enhances Nutrient Uptake, Yield, and NHX Gene Expression in Chinese Cabbage Under Salinity Stress
Source: Plants (Basel). 2025 Sep 2;14(17):2743. doi: 10.3390/plants14172743 (PMC12430363; doi:10.3390/plants14172743)
Supplement: Supplementary file 1 [file plants-14-02743-s001.zip › plants-3685186-supplementary.pdf]

Supplementary Figure:

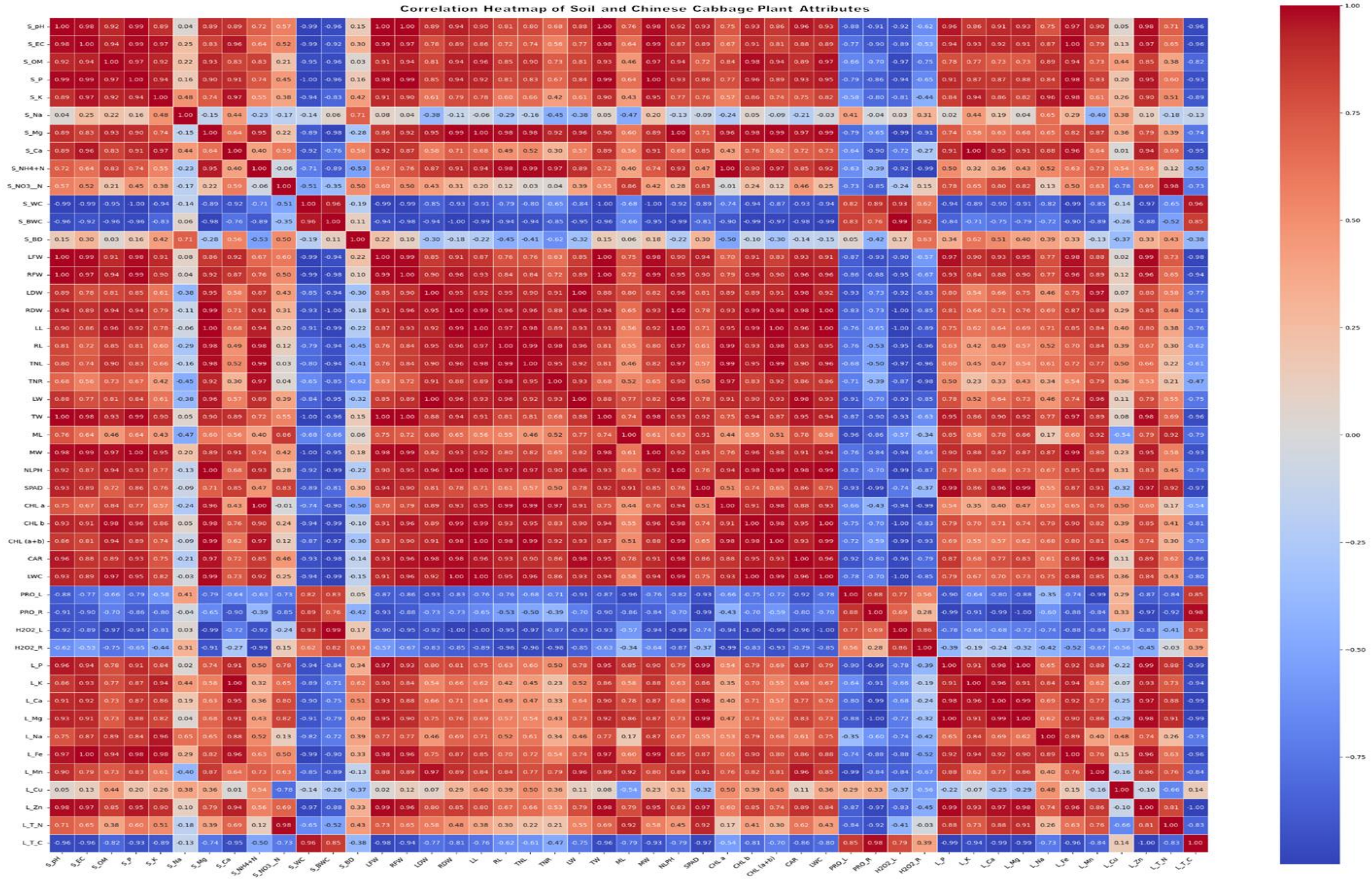

Figure S1. Heatmap of Pearson's correlation analysis of BC treatment on the soil properties, plant morphological, physiological, and biochemical parameters of Chinese cabbage exposed to salinity stress. Correlation values range from -1 (strong negative correlation) to +1 (strong positive correlation), with color gradients indicating the strength of correlation. (Top to bottom & Left to right- S-pH; S-EC; S-OM; S-P; S-K; S-Na; S-Mg; S-Ca; S-NH4+-N; S-NO3--N; S-WC; S-BWC; S-BD; LFW; RFW; LDW; RDW; LL; RL; TNL; TNR; LW; TW; ML; MW; NLPH; SPAD; CHL a; CHL b; CHL (a +b); CAR; LWC; PRO-L; PRO-R; H2O2-L; H2O2-R; L-P; L-K; L-Ca; L-Mg; L-Na; L-Fe; L-Mn; L-Cu; L-Zn; L-T-N; L-T-C).

Table S1. Details of NHX family transporters of Chinese cabbage.

Name of the gene	Gene ID	Location	LCDS	LP	NoC	TMDs	MW (kDa)	pI	Subcellular localization
BoNHX1	Bol032388	3040994.. 3044250	1608	535	09	11	59.35	7.62	PM
BoNHX2	Bol038549	173442..1 77417	1485	494	06	9	54.77	6.11	PM

Abbreviations: NoC, number of chromosomes; LP, length of protein sequences; LCDS, length of CDS sequences; pI, isoelectric point; TMDs, number of transmembrane domains; MW, molecular weight; PM, plasma membrane
